# Correction: Measurement tools and indicators for assessing nurturing care for early childhood development: A scoping review

**DOI:** 10.1371/journal.pgph.0001906

**Published:** 2023-05-02

**Authors:** Joshua Jeong, Lilia Bliznashka, Eileen Sullivan, Elizabeth Hentschel, Youngkwang Jeon, Kathleen L. Strong, Bernadette Daelmans

In the Funding section, a funder was omitted. The Financial Disclosure should read: This work was funded by the World Health Organization (grant # 2020/1047163 awarded to J.J.) with support from United States Agency for International Development (USAID) (Agreement GHA-G-00-09-0003). J.J. is supported, in part, by a Pathway to Independence Award from the Eunice Kennedy Shriver National Institute of Child Health and Human Development (K99HD105984). The funders had no role in study design, data extraction and analysis, decision to publish, or preparation of the manuscript.
