# Supplementary material for: Measurement tools and indicators for assessing nurturing care for early childhood development: A scoping review
Source: PLOS Glob Public Health. 2022 Apr 25;2(4):e0000373. doi: 10.1371/journal.pgph.0000373 (PMC10021181; doi:10.1371/journal.pgph.0000373)
Supplement: S1 Text — (DOCX) [file pgph.0000373.s002.docx]

**S1 Text. Search strategy.**

("Quality Indicators, Health Care"[Mesh:NoExp] OR indicator*[ti] OR measure*[ti] OR measuring[ti] OR metric*[ti] OR monitor*[ti] OR outcome*[ti] OR survey*[ti] OR tool[ti]) AND ("Child Development"[Mesh:NoExp] OR "Child Behavior"[Mesh:NoExp] OR child development*[tiab] OR childhood development*[tiab] OR children's development*[tiab] OR infant development*[tiab] OR neonatal development*[tiab])

AND ("Child Health"[Mesh] OR "Infant Health"[Mesh] OR child health[tiab] OR childrens health[tiab] OR children's health[tiab] OR infant health[tiab] OR neonatal health[tiab] OR maternal child health[tiab] OR maternal health[tiab] OR "Breast Feeding"[Mesh:NoExp] OR child nutrition[tiab] OR infant nutrition[tiab] OR malnutrition[tiab] OR complementary feeding[tiab] OR complementary food[tiab] OR infant feeding[tiab] OR infant and young child feeding[tiab] OR maternal nutrition[tiab] OR child feeding[tiab] OR feeding[tiab] OR micronutrient supplement*[tiab] OR nutrient supplement*[tiab] OR "Parenting"[Mesh] OR "Parent-Child Relations"[Mesh] OR parent-child[tiab] OR caregiver-child[tiab] OR parenting[tiab] OR caregiving[tiab] OR education[tiab] OR learning[tiab] OR safety[tiab] OR security[tiab] OR discipline[tiab] OR violence prevention[tiab])

AND (Infant[Mesh] OR "Child, Preschool"[Mesh] OR babies[tiab] OR baby[tiab] OR early childhood[tiab] OR young children*[tiab] OR infant*[tiab] OR infancy[tiab] OR neonatal[tiab] OR neonate[tiab] OR neonates[tiab] OR new born*[tiab] OR newborn*[tiab] OR perinatal[tiab] OR post natal[tiab] OR postnatal[tiab] OR pre school*[tiab] OR preschool*[tiab] OR toddler[tiab] OR toddlerhood[tiab] OR toddlers[tiab] OR 1 year of age[tiab] OR 1 year old*[tiab] OR 2 year old*[tiab] OR 2 years of age[tiab] OR 3 year old*[tiab] OR 3 years of age[tiab] OR age 0[tiab] OR age 1[tiab] OR age 2[tiab] OR age 3[tiab] OR age 4[tiab] OR age 5[tiab] OR age five[tiab] OR age four[tiab] OR age one[tiab] OR age three[tiab] OR age two[tiab] OR age zero[tiab] OR aged 0[tiab] OR aged 1[tiab] OR aged 2[tiab] OR aged 3[tiab] OR aged 4[tiab] OR aged 5[tiab] OR aged four[tiab] OR aged five[tiab] OR aged one[tiab] OR aged three[tiab] OR aged two[tiab] OR aged zero[tiab] OR ages 0[tiab] OR ages 1[tiab] OR ages 2[tiab] OR ages 3[tiab] OR ages 4[tiab] OR ages 5[tiab] OR ages five[tiab] OR ages four[tiab] OR ages one[tiab] OR ages three[tiab] OR ages two[tiab] OR one year old*[tiab] OR five year old*[tiab] OR four year old*[tiab] OR three year old*[tiab] OR two year old*[tiab] OR under 1 year[tiab] OR under 2 years[tiab] OR under 3 years[tiab] OR under 4 years[tiab] OR under 5 years[tiab])

AND ("Developing Countries"[Mesh] OR developing countr*[tiab] OR under developed countr*[tiab] OR lmic*[tiab] OR global[tiab] OR ((less developed[tiab] OR low income[tiab] OR lower income[tiab] OR middle income[tiab] OR resource poor[tiab] OR resource constrained[tiab] OR low resource[tiab] OR limited resource*[tiab] OR resource limited[tiab]) AND (country[tiab] OR countries[tiab] OR region[tiab] OR regions[tiab] OR setting*[tiab] OR area[tiab] OR areas[tiab])) OR "Africa South of the Sahara"[Mesh] OR "Egypt"[Mesh] OR "Tunisia"[Mesh] OR "Central America"[Mesh] OR "South America"[Mesh] OR "Latin America"[Mesh] OR "Cuba"[Mesh] OR "Dominica"[Mesh] OR "Dominican Republic"[Mesh] OR "Grenada"[Mesh] OR "Haiti"[Mesh] OR "Jamaica"[Mesh] OR "Saint Lucia"[Mesh] OR "Saint Vincent and the Grenadines"[Mesh] OR "Mexico"[Mesh] OR "Asia"[Mesh:NoExp] OR "Asia, Central"[Mesh] OR "Asia, Northern"[Mesh] OR "Asia, Southeastern"[Mesh] OR "Asia, Western"[Mesh] OR "China"[Mesh] OR "Mongolia"[Mesh] OR "Melanesia"[Mesh] OR "Samoa"[Mesh] OR "Micronesia"[Mesh:NoExp] OR "Albania"[Mesh] OR "Armenia"[Mesh] OR "Azerbaijan"[Mesh] OR "Bosnia and Herzegovina"[Mesh] OR "Bulgaria"[Mesh] OR "Georgia (Republic)"[Mesh] OR "Kosovo"[Mesh] OR "Moldova"[Mesh] OR "Montenegro"[Mesh] OR "Republic of Belarus"[Mesh] OR "Republic of North Macedonia"[Mesh] OR "Russia"[Mesh] OR "Serbia"[Mesh] OR "Ukraine"[Mesh] OR Afghan*[tiab] OR Africa[tiab] OR Albania*[tiab] OR Algeria*[tiab] OR American Samoa*[tiab] OR Angola*[tiab] OR Argentin*[tiab] OR Armenia*[tiab] OR Azerbaijan*[tiab] OR Bangladesh*[tiab] OR Belarus*[tiab] OR Belorus*[tiab] OR Beliz*[tiab] OR Benin*[tiab] OR Bhutan*[tiab] OR Bolivia*[tiab] OR Bosnia*[tiab] OR Botswan*[tiab] OR Brazil*[tiab] OR Bulgaria*[tiab] OR "Burkina Faso"[tiab] OR Burkinab*[tiab] OR Burund*[tiab] OR Cambodia*[tiab] OR Cameroon*[tiab] OR "Cape Verde"[tiab] OR "Cape Verdean"[tiab] OR "Central African Republic"[tiab] OR Chad*[tiab] OR China[tiab] OR Chinese[tiab] OR Colombia*[tiab] OR Comoros[tiab] OR Comorian[tiab] OR Congo[tiab] OR Congolese[tiab] OR "Costa Rica*"[tiab] OR "Côte d’Ivoire"[tiab] OR "Ivory Coast"[tiab] OR Ivorian[tiab] OR Cuba*[tiab] OR Djibouti*[tiab] OR Dominica*[tiab] OR "Dominican Republic"[tiab] OR Ecuador*[tiab] OR Egypt*[tiab] OR "El Salvador"[tiab] OR Salvadorian[tiab] OR "Equatorial Guinea"[tiab] OR Eritrea*[tiab] OR Ethiopia*[tiab] OR Eswatini[tiab] OR Fiji*[tiab] OR Gabon*[tiab] OR Gambia*[tiab] OR Gaza[tiab] OR Gazan[tiab] OR Georgia*[tiab] OR Ghana[tiab] OR Ghanaian[tiab] OR Grenad*[tiab] OR Guatemala*[tiab] OR Guinea[tiab] OR Guinean[tiab] OR Guyan*[tiab] OR Haiti*[tiab] OR Hercegovina[tiab] OR Herzegovina[tiab] OR Hondura*[tiab] OR India[tiab] OR Indian[tiab] OR Indonesia*[tiab] OR Iran*[tiab] OR Iraq*[tiab] OR Jamaica*[tiab] OR Jordan*[tiab] OR Kazakhstan*[tiab] OR Kenya[tiab] OR Kenyan[tiab] OR Kiribati[tiab] OR Kosov*[tiab] OR Kyrgy*[tiab] OR Laos[tiab] OR Laotian*[tiab] OR Lebanon[tiab] OR Lebanese[tiab] OR Lesotho[tiab] OR Liberia*[tiab] OR Libya*[tiab] OR Macedonia*[tiab] OR Madagasca*[tiab] OR Malawi*[tiab] OR Malaysia*[tiab] OR Maldives[tiab] OR Maldivian[tiab] OR Mali[tiab] OR Malian*[tiab] OR "Marshall Islands"[tiab] OR Mauritania*[tiab] OR Mauritian[tiab] OR Mayotte[tiab] OR Mexic*[tiab] OR Micronesia*[tiab] OR Moldov*[tiab] OR Mongolia*[tiab] OR Montenegr*[tiab] OR Morocc*[tiab] OR Mozambique[tiab] OR Mozambican[tiab] OR Myanmar[tiab] OR Namibia*[tiab] OR Nepal*[tiab] OR Nicaragua*[tiab] OR Niger*[tiab] OR Pakistan*[tiab] OR "Papua New Guinea"[tiab] OR Paraguay*[tiab] OR Peru*[tiab] OR Philippine*[tiab] OR Filipino*[tiab] OR Principe[tiab] OR Russia*[tiab] OR Rwanda*[tiab] OR "Saint Lucia"[tiab] OR "St Lucia"[tiab] OR "Saint Vincent" [tiab] OR "St Vincent"[tiab] OR Samoa*[tiab] OR "Sao Tome*"[tiab] OR Senegal*[tiab] OR Serbia*[tiab] OR "Sierra Leon*"[tiab] OR "Solomon Island*"[tiab] OR Somali*[tiab] OR "South Africa*"[tiab] OR "Sri Lanka*"[tiab] OR Sudan*[tiab] OR Suriname*[tiab] OR Swazi[tiab] OR Swaziland[tiab] OR Syria*[tiab] OR Tajik*[tiab] OR Tanzania*[tiab] OR Thailand[tiab] OR Thai[tiab] OR "Timor Leste"[tiab] OR Togo*[tiab] OR Tonga*[tiab] OR Tunisia*[tiab] OR Turkey[tiab] OR Turkish[tiab] OR Turkmenistan*[tiab] OR Tuvalu*[tiab] OR Uganda*[tiab] OR Ukrain*[tiab] OR Uzbek*[tiab] OR Vanuat*[tiab] OR Venezuela*[tiab] OR Viet Nam*[tiab] OR Vietnam*[tiab] OR "West Bank"[tiab] OR Yemen*[tiab] OR Zambia*[tiab] OR Zimbabwe*)
