# Supplementary material for: Measurement tools and indicators for assessing nurturing care for early childhood development: A scoping review
Source: PLOS Glob Public Health. 2022 Apr 25;2(4):e0000373. doi: 10.1371/journal.pgph.0000373 (PMC10021181; doi:10.1371/journal.pgph.0000373)
Supplement: S1 Table — (DOCX) [file pgph.0000373.s003.docx]

**S1 Table. Characteristics for each study included in scoping review on nurturing care indicators.**

| **Author** | **Year** | **Original study design** 1= pop survey (DHS/MICS, nationally representative) 2= household survey (not nationally rep, not program eval) 3= program evaluation 4= measurement  5= other (clinic-based health records; preschool; pooled trials) | **Youngest age at enrollment** 1= prenatal 2= 0-1 yr 3= 1-3 yr 4= 3-5 yr | **Sample size** 1= <100 2= 101-500 3= 501-1000 4= 1001-2000 5= >2000 | **Country** | **Early learning** | **Responsive caregiving** | **Early learning + responsive caregiving** | **Safety and security** | **Nutrition** | **Health** |
| --- | --- | --- | --- | --- | --- | --- | --- | --- | --- | --- | --- |
| Abessa et al. (2019) | 2019 | 3 | 2 | 2 | Ethiopia |  |  |  |  | X |  |
| Abimpaye, Dusabe, Nzabonimpa, Ashford, and Pisani (2019) | 2019 | 3 | 2 | 4 | Rwanda | X | X |  | X |  |  |
| Aboud (2007) | 2007 | 3 | 3 | 2 | Bangladesh | X | X | X |  | X | X |
| Aboud, Shafique, and Akhter (2009) | 2009 | 3 | 2 | 2 | Bangladesh |  | X |  |  | X |  |
| Aboud and Akhter (2011) | 2011 | 3 | 2 | 2 | Bangladesh |  | X | X |  | X | X |
| Aboud, Singla, Nahil, and Borisova (2013) | 2013 | 3 | 2 | 2 | Bangladesh |  |  | X |  | X | X |
| Addo et al. (2020) | 2020 | 3 | 2 | 5 | DRC |  |  |  |  | X |  |
| Ade, Gupta, Maliye, Deshmukh, and Garg (2010) | 2010 | 3 | 4 | 2 | India | X |  |  |  |  |  |
| Ahun, Aboud, Aryeetey, Colecraft, and Marquis (2017) | 2017 | 2 | 2 | 4 | Ghana | X |  |  |  | X | X |
| Andrew et al. (2020) | 2020 | 3 | 2 | 2 | India | X |  |  |  | X |  |
| Ara et al. (2019) | 2019 | 3 | 2 | 2 | Bangladesh |  |  |  |  | X | X |
| Aruna, Vazir, and Vidyasagar (2001) | 2001 | 2 | 3 | 2 | India |  | X |  |  | X |  |
| Attanasio et al. (2014) | 2014 | 3 | 3 | 4 | Colombia | X |  |  |  | X | X |
| Atukunda, Muhoozi, van den Broek, et al. (2019) | 2019 | 3 | 2 | 3 | Uganda |  |  |  |  | X | X |
| Atukunda, Muhoozi, Westerberg, and Iversen (2019) | 2019 | 3 | 2 | 2 | Uganda |  |  |  |  | X | X |
| Atukunda et al. (2020) | 2020 | 3 | 2 | 2 | Uganda |  |  |  |  | X | X |
| Baker-Henningham, Powell, Walker, and Grantham-McGregor (2005) | 2005 | 3 | 2 | 2 | Jamaica | X |  |  |  | X |  |
| Barros, Matijasevich, Santos, and Halpern (2010) | 2009 | 2 | 2 | 5 | Brazil | X |  |  |  | X |  |
| Bass et al. (2016) | 2016 | 3 | 3 | 2 | Uganda |  |  | X |  |  |  |
| Baydar and Akcinar (2018) | 2018 | 2 | 4 | 4 | Turkey |  | X |  | X |  |  |
| Berens et al. (2019) | 2019 | 4 | 3 | 2 | Bangladesh |  |  | X | X | X |  |
| Berkes, Raikes, Bouguen, and Filmer (2019) | 2019 | 3 | 3 | 5 | Cambodia | X |  |  |  |  |  |
| Bernal and Fernández (2013) | 2013 | 3 | 4 | 5 | Colombia |  |  |  |  | X |  |
| Bernal, Attanasio, Peña, and Vera-Hernández (2019) | 2019 | 3 | 2 | 5 | Colombia | X |  |  |  | X |  |
| Betancourt et al. (2020) | 2020 | 3 | 2 | 3 | Rwanda | X | X | X | X | X | X |
| Bhavnani et al. (2019) | 2019 | 3 | 3 | 1 | India | X |  |  |  | X |  |
| Bhopal et al. (2019) | 2019 | 3 | 3 | 4 | India |  | X | X | X | X | X |
| Binda, Figueroa-Leigh, and Olhaberry (2019) | 2019 | 3 | 2 | 2 | Chile | X | X |  | X | X |  |
| Black, Baqui, et al. (2004) | 2004 | 3 | 2 | 2 | Bangladesh |  |  | X |  | X |  |
| Black, Sazawal, et al. (2004) | 2004 | 3 | 2 | 2 | India |  |  | X |  | X | X |
| Black et al. (2007) | 2007 | 3 | 2 | 2 | Bangladesh | X |  |  |  | X |  |
| Black et al. (2019) | 2019 | 3 | 2 | 3 | India | X |  | X |  | X | X |
| Black et al. (2017) | 2017 | 1 | 2 | 5 | Multicountry | X |  |  |  |  |  |
| Blakstad et al. (2019) | 2019 | 3 | 2 | 2 | Tanzania |  |  |  |  | X | X |
| Bodeau-Livinec et al. (2019) | 2019 | 4 | 4 | 2 | Benin | X |  | X |  |  |  |
| Boivin et al. (2013b) | 2013 | 3 | 3 | 2 | Uganda |  | X | X |  | X | X |
| Boivin et al. (2013a) | 2013 | 3 | 3 | 2 | Uganda |  | X | X |  | X | X |
| Boivin et al. (2017) | 2017 | 4 | 4 | 1 | Uganda |  |  | X |  | X |  |
| Boo, Mateus, and Duryea (2018) | 2018 | 2 | 2 | 5 | Brazil | X |  | X | X | X |  |
| Bornstein and Putnick (2012) | 2012 | 1 | 2 | 5 | Multicountry | X |  |  | X |  |  |
| Bornstein, Putnick, Lansford, Deater-Deckard, and Bradley (2015) | 2015 | 1 | 2 | 5 | Multicountry | X |  |  |  |  |  |
| Bornstein, Putnick, Cote, Haynes, and Suwalsky (2015) | 2015 | 1 | 2 | 3 | Multicountry |  | X |  |  |  |  |
| Bougma, Aboud, Lemma, Frongillo, and Marquis (2018) | 2018 | 3 | 2 | 4 | Ethiopia |  |  | X |  | X |  |
| Brentani et al. (2021) | 2021 | 3 | 2 | 3 | Brazil | X |  |  |  | X |  |
| Bryant, Bryant, Williams, Ndambuki, and Erwin (2012) | 2012 | 3 | 2 | 5 | Kenya |  |  |  |  | X | X |
| Camargo-Figuera, Barros, Santos, Matijasevich, and Barros (2014) | 2014 | 2 | 1 | 5 | Brazil | X |  |  |  | X | X |
| Celhay, Martinez, and Vidal (2020) | 2020 | 1 | 2 | 5 | Bolivia |  |  |  |  | X |  |
| Chandna et al. (2020) | 2020 | 3 | 2 | 2 | Zimbabwe |  |  |  |  |  | X |
| Chang et al. (2015) | 2015 | 3 | 2 | 3 | Jamaica, Antigua, and St Lucia |  |  | X |  | X | X |
| Cheng, Lu, Archer, and Wang (2018) | 2018 | 2 | 2 | 1 | China |  | X |  |  |  |  |
| Chingono et al. (2018) | 2018 | 3 | 2 | 3 | Zimbabwe |  |  |  |  | X |  |
| Chung et al. (2020) | 2020 | 3 | 2 | 3 | Pakistan |  |  |  | X | X | X |
| Cooper et al. (2009) | 2009 | 3 | 1 | 2 | South Africa |  | X |  |  |  |  |
| Cuartas et al. (2019) | 2019 | 1 | 3 | 5 | 49 LMICs |  |  |  | X |  |  |
| da Rocha Neves, de Souza Morais, Teixeira, and Pinto (2016) | 2016 | 2 | 3 | 1 | Brazil | X |  | X |  | X | X |
| Dabar, Das, Nagesh, Yadav, and Mangal (2016) | 2016 | 2 | 2 | 3 | India | X |  |  |  | X | X |
| Dagvadorj et al. (2018) | 2018 | 2 | 3 | 2 | Mongolia |  |  |  |  | X | X |
| Davies et al. (2011) | 2011 | 2 | 2 | 2 | South Africa |  |  |  |  |  | X |
| de Castro et al. (2017) | 2017 | 1 | 2 | 5 | Mexico |  |  |  |  | X | X |
| de Moura et al. (2010) | 2010 | 2 | 2 | 5 | Brazil | X |  |  |  | X | X |
| Dearden et al. (2009) | 2009 | 1 | 3 | 1 | Vietnam |  | X |  |  |  |  |
| Diab, Isosävi, Qouta, Kuittinen, and Punamäki (2018) | 2018 | 2 | 2 | 3 | Gaza/Palestine |  |  |  |  |  | X |
| Diniz, DeSousa, Koller, and Volling (2016) | 2016 | 2 | 2 | 1 | Brazil |  | X |  |  |  |  |
| Do et al. (2020) | 2020 | 2 | 2 | 2 | Vietnam |  |  |  |  |  | X |
| Donald et al. (2018) | 2018 | 2 | 2 | 4 | South Africa |  | X |  | X |  |  |
| Donald et al. (2019) | 2019 | 2 | 2 | 4 | South Africa |  |  |  | X |  | X |
| Dora and Baydar (2020) | 2020 | 1 | 4 | 5 | Turkey |  |  |  | X |  |  |
| Drago et al. (2020) | 2020 | 2 | 2 | 2 | South Africa and Tanzania | X | X |  | X |  |  |
| du Toit, van der Linde, and Swanepoel (2020) | 2020 | 2 | 4 | 2 | South Africa |  |  |  |  |  | X |
| Duc (2016) | 2016 | 1 | 4 | 4 | Vietnam | X |  |  | X | X |  |
| Eickmann et al. (2007) | 2007 | 2 | 2 | 2 | Brazil |  |  | X |  | X |  |
| Ertem et al. (2006) | 2006 | 3 | 2 | 2 | Turkey | X |  | X |  |  | X |
| Familiar et al. (2018) | 2018 | 5 | 1 | 2 | Uganda |  |  | X |  | X |  |
| Fatori et al. (2020) | 2020 | 3 | 2 | 1 | Brazil | X |  |  |  |  |  |
| Feng et al. (2018) | 2018 | 2 | 2 | 2 | China |  |  |  |  |  | X |
| Fernald and Hidrobo (2011) | 2011 | 3 | 3 | 4 | Ecuador | X |  |  |  | X | X |
| Fernald, Jones-Smith, Ozer, Neufeld, and DiGirolamo (2008) | 2008 | 1 | 3 | 2 | Mexico |  |  |  |  | X |  |
| Fernald, Gertler, and Neufeld (2008) | 2008 | 3 | 3 | 5 | Mexico |  |  |  |  | X | X |
| Fernald, Kariger, Hidrobo, and Gertler (2012) | 2012 | 5 | 4 | 5 | Multicountry |  |  |  |  | X |  |
| Fink, McCoy, and Yousafzai (2020) | 2020 | 1 | 2 | 5 | Multicountry | X |  |  |  |  |  |
| Frith, Naved, Persson, Rasmussen, and Frongillo (2012) | 2012 | 3 | 1 | 2 | Bangladesh |  | X |  |  |  | X |
| Frongillo, Nguyen, et al. (2017) | 2017 | 3 | 2 | 5 | Bangladesh | X | X |  |  | X | X |
| Frongillo, Kulkarni, Basnet, and de Castro (2017) | 2017 | 1 | 4 | 5 | 26 LMICs | X |  |  | X | X |  |
| Galasso, Weber, Stewart, Ratsifandrihamanana, and Fernald (2019) | 2019 | 3 | 2 | 5 | Madagascar | X |  |  |  | X | X |
| Geletu, Lelisa, and Baye (2019) | 2019 | 3 | 2 | 2 | Ethiopia |  |  |  |  | X | X |
| Gelli et al. (2018) | 2018 | 3 | 2 | 4 | Malawi |  |  |  |  | X |  |
| Gelli et al. (2020) | 2020 | 3 | 2 | 4 | Malawi |  |  |  |  | X |  |
| Gladstone et al. (2019) | 2019 | 3 | 3 | 4 | Zimbabwe |  |  |  |  | X | X |
| Grantham-McGregor et al. (2020) | 2020 | 3 | 2 | 2 | India | X | X |  |  | X | X |
| Hamadani et al. (2019) | 2019 | 3 | 2 | 5 | Bangladesh | X |  |  |  | X |  |
| Hamadani et al. (2010) | 2010 | 4 | 3 | 3 | Bangladesh | X |  |  |  | X | X |
| Hamadani et al. (2014) | 2014 | 3 | 2 | 5 | Bangladesh |  |  | X |  | X |  |
| Handal, Lozoff, Breilh, and Harlow (2007a) | 2007 | 2 | 2 | 2 | Ecuador | X |  |  |  | X | X |
| Handal, Lozoff, Breilh, and Harlow (2007b) | 2007 | 2 | 3 | 2 | Ecuador | X |  |  |  | X |  |
| Handal et al. (2017) | 2017 | 4 | 2 | 1 | Ecuador |  | X |  |  |  |  |
| Handal, Lozoff, Breilh, and Harlow (2007c) | 2007 | 2 | 2 | 2 | Ecuador | X |  |  |  | X | X |
| Hanieh et al. (2014) | 2014 | 3 | 1 | 3 | Vietnam |  |  |  |  | X | X |
| Hartinger et al. (2011) | 2011 | 3 | 2 | 3 | Peru |  |  |  |  | X | X |
| Hartinger et al. (2016) | 2016 | 3 | 2 | 3 | Peru |  |  |  |  | X | X |
| Hartinger et al. (2017) | 2017 | 3 | 2 | 3 | Peru |  |  |  |  | X | X |
| Hollowell, Belem, Swigart, Murray, and Hill (2020) | 2020 | 2 | 2 | 3 | Burkina Faso | X |  |  |  |  |  |
| Hua et al. (2016) | 2016 | 2 | 4 | 5 | China | X |  |  |  |  |  |
| Huiracocha-Tutiven et al. (2019) | 2019 | 2 | 3 | 2 | Ecuador |  |  |  |  | X |  |
| Huynh, Tran, Nguyen, Berde, and Low (2018) | 2018 | 3 | 1 | 2 | Vietnam |  |  | X |  | X |  |
| Ip et al. (2018) | 2018 | 4 | 4 | 3 | China | X |  |  |  |  |  |
| Jensen, Tofail, Haque, Petri, and Nelson (2019) | 2019 | 2 | 2 | 2 | Bangladesh | X |  |  |  | X | X |
| Jeong, Bhatia, and Fink (2018) | 2018 | 1 | 2 | 5 | 31 LMICs | X |  |  | X | X | X |
| Jeong, Siyal, and Yousafzai (2019) | 2019 | 2 | 2 | 1 | Pakistan | X | X |  |  |  |  |
| Jeong, McCoy, Yousafzai, Salhi, and Fink (2016) | 2016 | 1 | 4 | 5 | Multicountry | X |  |  |  | X |  |
| Jeong, Adhia, Bhatia, McCoy, and Yousafzai (2020) | 2020 | 1 | 4 | 5 | Multicountry | X |  |  | X |  |  |
| Kang, Aguayo, Campbell, and West (2018) | 2018 | 1 | 4 | 5 | Bangladesh, Bhutan, Nepal, Pakistan | X |  |  | X | X | X |
| Karabekiroglu et al. (2013) | 2013 | 1 | 2 | 4 | Turkey | X |  |  | X |  |  |
| Kariger et al. (2012) | 2012 | 4 | 2 | 5 | Multicountry | X | X |  | X |  |  |
| Kashala-Abotnes et al. (2018) | 2018 | 2 | 3 | 2 | DRC | X |  |  | X | X | X |
| Khandelwal et al. (2020) | 2020 | 2 | 2 | 2 | India |  |  |  |  | X | X |
| Khowaja et al. (2015) | 2015 | 3 | 3 | 1 | Pakistan |  |  |  | X |  |  |
| Knauer, Ozer, Dow, and Fernald (2017) | 2017 | 3 | 2 | 4 | Mexico |  |  | X |  |  |  |
| Knauer, Jakiela, Ozier, Aboud, and Fernald (2019) | 2019 | 3 | 3 | 2 | Kenya | X | X |  |  | X |  |
| Koshy et al. (2020) | 2020 | 2 | 2 | 2 | India |  |  |  |  | X |  |
| Koura et al. (2013) | 2013 | 3 | 2 | 2 | Benin |  |  | X |  |  |  |
| Kumar, Aggarwal, Kaur, and Iyengar (1997) | 1997 | 2 | 2 | 5 | India | X |  |  |  | X | X |
| Kumwenda et al. (2018) | 2018 | 3 | 2 | 2 | Malawi | X |  |  |  | X |  |
| Kvestad et al. (2015) | 2015 | 3 | 2 | 2 | India | X | X |  |  | X | X |
| Lahti et al. (2019) | 2019 | 2 | 1 | 3 | Palestine |  | X |  |  |  | X |
| Larson et al. (2017) | 2017 | 3 | 2 | 5 | India | X |  |  |  | X | X |
| Larson, Young, et al. (2018) | 2018 | 3 | 2 | 5 | India | X |  |  |  | X | X |
| Larson, Martorell, and Bauer (2018) | 2018 | 3 | 3 | 4 | India | X |  |  |  | X |  |
| Laurenzi et al. (2020) | 2020 | 3 | 4 | 2 | Kenya |  |  |  | X |  | X |
| le Roux et al. (2018) | 2018 | 5 | 1 | 3 | South Africa | X |  |  | X | X |  |
| Li, Jiang, Li, and Lu (2018) | 2018 | 1 | 3 | 5 | China | X |  |  |  | X |  |
| le Roux et al. (2018) | 2019 | 2 | 2 | 1 | China |  | X |  |  |  |  |
| Lima et al. (2004) | 2004 | 5 | 2 | 2 | Brazil |  |  | X |  | X | X |
| Locks et al. (2019) | 2019 | 3 | 2 | 5 | DRC |  |  |  |  | X |  |
| Boo et al. (2018) | 2018 | 2 | 2 | 5 | Brazil | X |  | X | X | X |  |
| Lozoff et al. (2010) | 2010 | 3 | 2 | 2 | Chile |  |  | X |  | X | X |
| Lu et al. (2020) | 2020 | 1 | 4 | 5 | LMICs | X |  |  |  |  |  |
| Luo et al. (2015) | 2015 | 3 | 2 | 4 | China |  |  |  |  | X | X |
| Luo et al. (2017) | 2017 | 3 | 2 | 3 | China | X |  |  |  | X |  |
| Luo, Emmers, Warrinnier, Rozelle, and Sylvia (2019) | 2019 | 3 | 2 | 2 | China | X |  |  |  | X | X |
| Luoto et al. (2021) | 2019 | 3 | 2 | 4 | Kenya | X |  | X |  | X | X |
| MacGinty et al. (2020) | 2020 | 2 | 2 | 3 | South Africa |  |  |  | X | X | X |
| Malhi, Menon, Bharti, and Sidhu (2018) | 2018 | 2 | 3 | 2 | India | X | X |  |  |  |  |
| Manu, Ewerling, Barros, and Victora (2019) | 2019 | 1 | 4 | 5 | 35 Countries | X |  |  |  |  |  |
| Martinez, Cardona, et al. (2018) | 2018 | 3 | 2 | 2 | Guatemala | X |  |  |  | X |  |
| Martinez, Webb, et al. (2018) | 2018 | 3 | 2 | 2 | Guatemala | X |  |  |  | X |  |
| Maselko et al. (2019) | 2019 | 3 | 2 | 4 | Pakistan | X | X |  |  | X |  |
| Mathieu et al. (2020) | 2020 | 2 | 3 | 1 | Dominican Republic | X |  |  |  |  |  |
| McCoy et al. (2017) | 2017 | 4 | 3 | 5 | Tanzania | X |  |  |  | X | X |
| McDonald et al. (2013) | 2013 | 3 | 2 | 2 | Tanzania |  |  |  |  | X | X |
| Mazzucchelli et al. (2018) | 2018 | 3 | 2 | 3 | Zimbabwe |  |  |  |  | X | X |
| Mebrahtu et al. (2019) | 2019 | 3 | 2 | 3 | Zimbabwe |  |  |  |  | X | X |
| Miller et al. (2020) | 2020 | 3 | 2 | 3 | Zimbabwe |  |  |  |  | X | X |
| Menon 2016 | 2016 | 3 | 2 | 3 | Bangladesh and Vietnam |  |  |  |  | X |  |
| Miller, Murray, Thomson, and Arbour (2016) | 2016 | 1 | 4 | 5 | 15 LMICs | X |  |  |  | X |  |
| Miller et al. (2020) | 2020 | 3 | 4 | 2 | Madagascar | X |  |  |  | X |  |
| Mireku, Cot, Massougbodji, and Bodeau-Livinec (2020) | 2020 | 3 | 2 | 3 | Benin |  |  | X |  | X |  |
| Morris et al. (2012) | 2012 | 3 | 2 | 2 | Uganda | X | X |  |  |  |  |
| Muhoozi, Atukunda, Mwadime, Iversen, and Westerberg (2016) | 2016 | 3 | 2 | 3 | Uganda |  |  |  |  | X | X |
| Muhoozi et al. (2017) | 2017 | 3 | 2 | 3 | Uganda |  |  |  |  | X | X |
| Murray et al. (2016) | 2016 | 3 | 3 | 1 | South Africa |  | X |  |  |  |  |
| Nahar et al. (2009) | 2009 | 3 | 2 | 1 | Bangladesh |  |  |  |  | X |  |
| Nahar, Hossain, Hamadani, Ahmed, Huda, et al. (2012) | 2012 | 3 | 2 | 3 | Bangladesh | X |  |  |  | X |  |
| Nahar, Hossain, Hamadani, Ahmed, Grantham-McGregor, et al. (2012) | 2012 | 3 | 2 | 3 | Bangladesh | X |  | X |  | X |  |
| Nahar et al. (2015) | 2015 | 3 | 2 | 3 | Bangladesh | X |  | X |  | X |  |
| Nahar et al. (2020) | 2020 | 2 | 2 | 2 | Bangladesh |  |  |  |  | X |  |
| Obradović, Yousafzai, Finch, and Rasheed (2016) | 2016 | 3 | 2 | 4 | Pakistan |  | X | X |  | X |  |
| Ocansey et al. (2019) | 2019 | 3 | 4 | 3 | Ghana |  |  | X |  |  |  |
| Olney, Leroy, Bliznashka, and Ruel (2019) | 2019 | 3 | 2 | 5 | Burundi |  |  |  |  | X | X |
| Özler et al. (2018) | 2018 | 3 | 4 | 5 | Malawi | X |  |  |  | X |  |
| Pace, Lee, and Grogan-Kaylor (2019) | 2019 | 1 | 4 | 5 | Multicountry |  |  |  | X |  |  |
| Park et al. (2016) | 2016 | 3 | 1 | 2 | the Philippines |  |  |  |  | X | X |
| Pearson et al. (2019) | 2019 | 2 | 4 | 1 | Brazil | X |  |  |  |  |  |
| Perez et al. (2005) | 2005 | 3 | 2 | 1 | South Africa |  | X |  |  | X | X |
| Pitchik et al. (2018) | 2018 | 3 | 3 | 2 | Tanzania | X |  |  | X | X | X |
| Ponguta et al. (2020) | 2020 | 3 | 3 | 2 | Lebanon | X | X |  | X | X |  |
| Powell, Baker-Henningham, Walker, Gernay, and Grantham-McGregor (2004) | 2004 | 3 | 2 | 2 | Jamaica | X |  |  |  | X |  |
| Prado et al. (2020) | 2020 | 3 | 2 | 3 | Malawi |  |  | X |  | X | X |
| Quevedo et al. (2012) | 2012 | 2 | 2 | 2 | Brazil |  |  |  |  | X | X |
| Rao et al. (2020) | 2020 | 4 | 4 | 5 | Multicountry | X |  |  |  | X |  |
| Rasheed and Yousafzai (2015) | 2015 | 4 | 3 | 4 | Pakistan | X | X |  |  | X |  |
| L. A. Rempel, Rempel, Khuc, and Vui (2017) | 2017 | 3 | 1 | 3 | Vietnam |  | X |  |  | X |  |
| J. K. Rempel, Rempel, Hoa, Vui, and Long (2020) | 2020 | 3 | 1 | 3 | Vietnam |  |  |  |  | X |  |
| Ribe, Svensen, Lyngmo, Mduma, and Hinderaker (2018) | 2018 | 2 | 2 | 2 | Tanzania |  | X |  | X | X | X |
| Rockers et al. (2016) | 2016 | 3 | 2 | 3 | Zambia | X |  |  |  | X | X |
| Rockers et al. (2018) | 2018 | 3 | 2 | 2 | Zambia | X |  |  |  | X | X |
| Rodriguez et al. (2018) | 2018 | 3 | 1 | 1 | South Africa |  |  |  | X |  |  |
| Roopnarine, Jin, and Krishnakumar (2014) | 2014 | 2 | 4 | 2 | Guyana |  |  |  | X |  |  |
| Rubio-Codina, Attanasio, Meghir, Varela, and Grantham-McGregor (2015) | 2015 | 3 | 2 | 4 | Colombia | X |  |  |  | X | X |
| Rubio-Codina and Grantham-McGregor (2019) | 2019 | 3 | 2 | 4 | Colombia | X |  |  |  | X |  |
| Sajedi, Habibi, Hatamizadeh, Shahshahanipour, and Malek Afzali (2018) | 2019 | 2 | 2 | 2 | Iran | X |  |  |  |  |  |
| Salhi et al. (2021) | 2021 | 1 | 4 | 5 | Multicountry | X |  |  | X |  |  |
| Samanta et al. (2017) | 2017 | 2 | 4 | 2 | India | X |  |  |  |  |  |
| Sangtawesin, Singarj, and Kanjanapattanakul (2011) | 2011 | 5 | 3 | 1 | Thailand |  |  |  |  | X |  |
| Sania et al. (2019) | 2019 | 5 | 1 | 5 | Multicountry |  |  |  |  | X | X |
| Sarkar and Sarkar (2017) | 2017 | 1 | 2 | 5 | India | X |  |  |  | X | X |
| Scharf et al. (2018) | 2018 | 2 | 2 | 4 | Multicountry |  |  |  |  | X | X |
| Scherer et al. (2019) | 2019 | 3 | 1 | 3 | Pakistan |  | X | X |  | X |  |
| Schneider et al. (2021) | 2021 | 4 | 3 | 2 | Brazil |  | X |  |  |  |  |
| Servili et al. (2010) | 2010 | 2 | 1 | 2 | Ethiopia |  |  |  | X | X | X |
| Shattnawi and Al-Ali (2019) | 2019 | 3 | 2 | 1 | Jordan |  |  |  |  | X | X |
| Shi, Li, Fang, Zhang, and Wang (2020) | 2020 | 3 | 2 | 2 | China |  |  |  |  | X | X |
| Sikander et al. (2019) | 2019 | 3 | 1 | 4 | Pakistan |  | X | X | X | X | X |
| Sincovich et al. (2020) | 2020 | 4 | 4 | 5 | Multicountry | X |  |  |  | X |  |
| Singla, Kumbakumba, and Aboud (2015) | 2015 | 3 | 3 | 2 | Uganda |  |  | X |  | X | X |
| Smuts et al. (2019) | 2019 | 3 | 2 | 3 | South Africa |  |  |  |  | X | X |
| Struyf et al. (2020) | 2020 | 2 | 2 | 3 | Malawi |  |  |  |  | X |  |
| Surkan et al. (2015) | 2015 | 3 | 2 | 3 | Nepal |  |  |  |  | X | X |
| Tessier et al. (2009) | 2009 | 3 | 2 | 2 | Colombia |  |  | X |  |  | X |
| Till et al. (2019) | 2019 | 2 | 1 | 1 | Costa Rica |  | X | X |  |  |  |
| Tofail et al. (2013) | 2013 | 3 | 2 | 2 | Bangladesh | X |  |  |  | X | X |
| Tomlinson et al. (2020) | 2020 | 3 | 1 | 2 | South Africa |  | X |  |  |  |  |
| Tran, Luchters, and Fisher (2017) | 2017 | 1 | 4 | 5 | Multicountry | X |  |  | X |  |  |
| Tumwine et al. (2018) | 2018 | 3 | 2 | 3 | Burkina Faso and Uganda |  |  |  |  | X |  |
| Uwemedimo, Howlader, and Pierret (2017) | 2017 | 5 | 2 | 1 | Dominican Republic |  | X |  | X | X |  |
| Vazir et al. (2013) | 2013 | 3 | 1 | 3 | India |  |  |  |  | X | X |
| Wang et al. (2020) | 2020 | 2 | 3 | 1 | China |  |  |  |  | X |  |
| Weber et al. (2019) | 2019 | 4 | 2 | 5 | Multicountry |  |  |  |  | X | X |
| Wedderburn et al. (2019) | 2019 | 2 | 2 | 4 | South Africa |  |  |  |  | X | X |
| Wei et al. (2015) | 2015 | 2 | 2 | 5 | China | X |  |  |  |  |  |
| Wei, Zhang, Zhang, Luo, and Wang (2018) | 2018 | 2 | 2 | 5 | China |  |  |  | X |  |  |
| Weisleder et al. (2018) | 2018 | 3 | 3 | 3 | Brazil | X | X |  | X |  |  |
| Wolf and McCoy (2019) | 2019 | 3 | 4 | 5 | Ghana | X |  |  |  |  |  |
| Worku, Abessa, Wondafrash, Vanvuchelen, et al. (2018) | 2018 | 2 | 2 | 4 | Ethiopia | X |  |  | X | X |  |
| Worku, Abessa, Wondafrash, Lemmens, et al. (2018) | 2018 | 3 | 2 | 1 | Ethiopia |  |  |  |  | X |  |
| Xing, Wang, and Wang (2018) | 2018 | 2 | 4 | 2 | China |  |  |  | X |  |  |
| Yang et al. (2020) | 2020 | 2 | 2 | 2 | China |  |  |  |  | X |  |
| Ye et al. (2019) | 2019 | 5 | 1 | 5 | China |  |  |  |  | X | X |
| Yousafzai, Rasheed, Rizvi, Armstrong, and Bhutta (2014) | 2014 | 3 | 2 | 4 | Pakistan |  |  |  |  | X | X |
| Yousafzai, Rasheed, Rizvi, Armstrong, and Bhutta (2015) | 2015 | 3 | 2 | 4 | Pakistan | X | X | X |  |  |  |
| Yousafzai et al. (2016) | 2016 | 3 | 2 | 4 | Pakistan | X | X | X |  | X | X |
| Yue et al. (2019) | 2019 | 3 | 3 | 4 | China | X |  |  |  |  |  |
| J. Zhang et al. (2018) | 2018 | 2 | 2 | 5 | China | X |  |  |  | X | X |
| S. Zhang et al. (2018) | 2018 | 2 | 2 | 4 | China | X |  |  | X |  |  |
| Z. Zhang et al. (2018) | 2018 | 3 | 1 | 2 | Vietnam |  |  | X |  | X |  |
| Zhong and Luo (2020) | 2020 | 2 | 2 | 4 | China | X |  |  |  |  | X |
| Zhong, He, Gao, Wang, and Luo (2020) | 2020 | 2 | 2 | 4 | China | X |  |  |  |  |  |
| Zhong, He, Chen, and Luo (2020) | 2020 | 2 | 2 | 4 | China | X |  |  | X |  |  |
| Zhou et al. (2019) | 2019 | 3 | 2 | 5 | China | X |  |  | X | X |  |

**References for each study included in the review**

Abessa, T. G., Worku, B. N., Wondafrash, M., Girma, T., Valy, J., Lemmens, J., . . . Granitzer, M. (2019). Effect of play-based family-centered psychomotor/psychosocial stimulation on the development of severely acutely malnourished children under six in a low-income setting: a randomized controlled trial. *BMC Pediatr, 19*(1), 336. doi:10.1186/s12887-019-1696-z

Abimpaye, M., Dusabe, C., Nzabonimpa, J. P., Ashford, R., & Pisani, L. (2019). Improving parenting practices and development for young children in Rwanda: Results from a randomized control trial. *International Journal of Behavioral Development, 44*(3), 205-215. doi:10.1177/0165025419861173

Aboud, F. E. (2007). Evaluation of an early childhood parenting programme in rural Bangladesh. *J Health Popul Nutr, 25*(1), 3-13.

Aboud, F. E., & Akhter, S. (2011). A cluster-randomized evaluation of a responsive stimulation and feeding intervention in bangladesh. *Pediatrics, 127*(5), e1191-1197. doi:10.1542/peds.2010-2160

Aboud, F. E., Shafique, S., & Akhter, S. (2009). A responsive feeding intervention increases children's self-feeding and maternal responsiveness but not weight gain. *J Nutr, 139*(9), 1738-1743. doi:10.3945/jn.109.104885

Aboud, F. E., Singla, D. R., Nahil, M. I., & Borisova, I. (2013). Effectiveness of a parenting program in Bangladesh to address early childhood health, growth and development. *Soc Sci Med, 97*, 250-258. doi:10.1016/j.socscimed.2013.06.020

Addo, O. Y., Tripp, K., Nanama, S., Albert, B., Sandalinas, F., Nanema, A., . . . Locks, L. M. (2020). An Integrated Infant and Young Child Feeding and Small-Quantity Lipid-based Nutrient Supplementation Program Is Associated with Improved Gross Motor and Communication Scores of Children 6-18 Months in the Democratic Republic of Congo. *J Pediatr, 222*, 154-163. doi:10.1016/j.jpeds.2020.01.023

Ade, A., Gupta, S. S., Maliye, C., Deshmukh, P. R., & Garg, B. S. (2010). Effect of improvement of pre-school education through Anganwadi center on intelligence and development quotient of children. *Indian J Pediatr, 77*(5), 541-546. doi:10.1007/s12098-010-0056-7

Ahun, M. N., Aboud, F. E., Aryeetey, R., Colecraft, E., & Marquis, G. S. (2017). Child development in rural Ghana: Associations between cognitive/language milestones and indicators of nutrition and stimulation of children under two years of age. *Can J Public Health, 108*(5-6), e578-e585. doi:10.17269/cjph.108.5875

Andrew, A., Attanasio, O., Augsburg, B., Day, M., Grantham-McGregor, S., Meghir, C., . . . Rubio-Codina, M. (2020). Effects of a scalable home-visiting intervention on child development in slums of urban India: evidence from a randomised controlled trial. *J Child Psychol Psychiatry, 61*(6), 644-652. doi:10.1111/jcpp.13171

Ara, G., Khanam, M., Papri, N., Nahar, B., Kabir, I., Sanin, K. I., . . . Dibley, M. J. (2019). Peer Counseling Promotes Appropriate Infant Feeding Practices and Improves Infant Growth and Development in an Urban Slum in Bangladesh: A Community-Based Cluster Randomized Controlled Trial. *Curr Dev Nutr, 3*(7), nzz072. doi:10.1093/cdn/nzz072

Aruna, M., Vazir, S., & Vidyasagar, P. (2001). Child rearing and positive deviance in the development of preschoolers: A Micro Analysis. *Indian pediatrics, 38*(4), 332-339.

Attanasio, O. P., Fernández, C., Fitzsimons, E. O., Grantham-McGregor, S. M., Meghir, C., & Rubio-Codina, M. (2014). Using the infrastructure of a conditional cash transfer program to deliver a scalable integrated early child development program in Colombia: cluster randomized controlled trial. *BMJ, 349*, g5785. doi:10.1136/bmj.g5785

Atukunda, P., Muhoozi, G. K., Diep, L. M., Berg, J. P., Westerberg, A. C., & Iversen, P. O. (2020). The association of urine markers of iodine intake with development and growth among children in rural Uganda: a secondary analysis of a randomised education trial. *Public Health Nutr*, 1-10. doi:10.1017/S1368980020001603

Atukunda, P., Muhoozi, G. K. M., van den Broek, T. J., Kort, R., Diep, L. M., Kaaya, A. N., . . . Westerberg, A. C. (2019). Child development, growth and microbiota: follow-up of a randomized education trial in Uganda. *J Glob Health, 9*(1), 010431. doi:10.7189/jogh-09-010431

Atukunda, P., Muhoozi, G. K. M., Westerberg, A. C., & Iversen, P. O. (2019). Nutrition, Hygiene and Stimulation Education for Impoverished Mothers in Rural Uganda: Effect on Maternal Depression Symptoms and Their Associations to Child Development Outcomes. *Nutrients, 11*(7). doi:10.3390/nu11071561

Baker-Henningham, H., Powell, C., Walker, S., & Grantham-McGregor, S. (2005). The effect of early stimulation on maternal depression: a cluster randomised controlled trial. *Arch Dis Child, 90*(12), 1230-1234. doi:10.1136/adc.2005.073015

Barros, A. J., Matijasevich, A., Santos, I. S., & Halpern, R. (2010). Child development in a birth cohort: effect of child stimulation is stronger in less educated mothers. *Int J Epidemiol, 39*(1), 285-294. doi:10.1093/ije/dyp272

Bass, J. K., Nakasujja, N., Familiar-Lopez, I., Sikorskii, A., Murray, S. M., Opoka, R., . . . Boivin, M. J. (2016). Association of caregiver quality of care with neurocognitive outcomes in HIV-affected children aged 2-5 years in Uganda. *AIDS Care, 28 Suppl 1*(sup1), 76-83. doi:10.1080/09540121.2016.1146215

Baydar, N., & Akcinar, B. (2018). Reciprocal Relations between the Trajectories of Mothers' Harsh Discipline, Responsiveness and Aggression in Early Childhood. *J Abnorm Child Psychol, 46*(1), 83-97. doi:10.1007/s10802-017-0280-y

Berens, A. E., Kumar, S., Tofail, F., Jensen, S. K. G., Alam, M., Haque, R., . . . Nelson, C. A., 3rd. (2019). Cumulative psychosocial risk and early child development: validation and use of the Childhood Psychosocial Adversity Scale in global health research. *Pediatr Res, 86*(6), 766-775. doi:10.1038/s41390-019-0431-7

Berkes, J., Raikes, A., Bouguen, A., & Filmer, D. (2019). Joint roles of parenting and nutritional status for child development: Evidence from rural Cambodia. *Dev Sci, 22*(5), e12874. doi:10.1111/desc.12874

Bernal, R., Attanasio, O., Peña, X., & Vera-Hernández, M. (2019). The effects of the transition from home-based childcare to childcare centers on children's health and development in Colombia. *Early Child Res Q, 47*, 418-431. doi:10.1016/j.ecresq.2018.08.005

Bernal, R., & Fernández, C. (2013). Subsidized childcare and child development in Colombia: effects of Hogares Comunitarios de Bienestar as a function of timing and length of exposure. *Soc Sci Med, 97*, 241-249. doi:10.1016/j.socscimed.2012.10.029

Betancourt, T. S., Jensen, S. K. G., Barnhart, D. A., Brennan, R. T., Murray, S. M., Yousafzai, A. K., . . . Kamurase, A. (2020). Promoting parent-child relationships and preventing violence via home-visiting: a pre-post cluster randomised trial among Rwandan families linked to social protection programmes. *BMC Public Health, 20*(1), 621. doi:10.1186/s12889-020-08693-7

Bhavnani, S., Mukherjee, D., Dasgupta, J., Verma, D., Parameshwaran, D., Divan, G., . . . Patel, V. (2019). Development, feasibility and acceptability of a gamified cognitive DEvelopmental assessment on an E-Platform (DEEP) in rural Indian pre-schoolers - a pilot study. *Glob Health Action, 12*(1), 1548005. doi:10.1080/16549716.2018.1548005

Bhopal, S., Roy, R., Verma, D., Kumar, D., Avan, B., Khan, B., . . . Kirkwood, B. R. (2019). Impact of adversity on early childhood growth & development in rural India: Findings from the early life stress sub-study of the SPRING cluster randomised controlled trial (SPRING-ELS). *PLoS One, 14*(1), e0209122. doi:10.1371/journal.pone.0209122

Binda, V., Figueroa-Leigh, F., & Olhaberry, M. (2019). Antenatal and postnatal depressive symptoms: Association with quality of mother-infant interaction. *Infant Behav Dev, 57*, 101386. doi:10.1016/j.infbeh.2019.101386

Black, M. M., Baqui, A. H., Zaman, K., Ake Persson, L., El Arifeen, S., Le, K., . . . Black, R. E. (2004). Iron and zinc supplementation promote motor development and exploratory behavior among Bangladeshi infants. *Am J Clin Nutr, 80*(4), 903-910. doi:10.1093/ajcn/80.4.903

Black, M. M., Baqui, A. H., Zaman, K., McNary, S. W., Le, K., Arifeen, S. E., . . . Black, R. E. (2007). Depressive symptoms among rural Bangladeshi mothers: implications for infant development. *J Child Psychol Psychiatry, 48*(8), 764-772. doi:10.1111/j.1469-7610.2007.01752.x

Black, M. M., Sazawal, S., Black, R. E., Khosla, S., Kumar, J., & Menon, V. (2004). Cognitive and motor development among small-for-gestational-age infants: impact of zinc supplementation, birth weight, and caregiving practices. *Pediatrics, 113*(5), 1297-1305. doi:10.1542/peds.113.5.1297

Black, M. M., Walker, S. P., Fernald, L. C. H., Andersen, C. T., DiGirolamo, A. M., Lu, C., . . . Grantham-McGregor, S. (2017). Early childhood development coming of age: science through the life course. *The Lancet, 389*(10064), 77-90. doi:10.1016/S0140-6736(16)31389-7

Black, M. M., Yimgang, D. P., Hurley, K. M., Harding, K. B., Fernandez-Rao, S., Balakrishna, N., . . . Nair, K. M. (2019). Mechanisms linking height to early child development among infants and preschoolers in rural India. *Dev Sci, 22*(5), e12806. doi:10.1111/desc.12806

Blakstad, M. M., Smith, E. R., Etheredge, A., Locks, L. M., McDonald, C. M., Kupka, R., . . . Duggan, C. P. (2019). Nutritional, Socioeconomic, and Delivery Characteristics Are Associated with Neurodevelopment in Tanzanian Children. *J Pediatr, 207*, 71-79.e78. doi:10.1016/j.jpeds.2018.10.066

Bodeau-Livinec, F., Davidson, L. L., Zoumenou, R., Massougbodji, A., Cot, M., & Boivin, M. J. (2019). Neurocognitive testing in West African children 3-6 years of age: Challenges and implications for data analyses. *Brain Res Bull, 145*, 129-135. doi:10.1016/j.brainresbull.2018.04.001

Boivin, M. J., Bangirana, P., Nakasujja, N., Page, C. F., Shohet, C., Givon, D., . . . Klein, P. S. (2013a). A year-long caregiver training program improves cognition in preschool Ugandan children with human immunodeficiency virus. *J Pediatr, 163*(5), 1409-1416.e1401. doi:10.1016/j.jpeds.2013.06.055

Boivin, M. J., Bangirana, P., Nakasujja, N., Page, C. F., Shohet, C., Givon, D., . . . Klein, P. S. (2013b). A year-long caregiver training program to improve neurocognition in preschool Ugandan HIV-exposed children. *J Dev Behav Pediatr, 34*(4), 269-278. doi:10.1097/DBP.0b013e318285fba9

Boivin, M. J., Weiss, J., Chhaya, R., Seffren, V., Awadu, J., Sikorskii, A., & Giordani, B. (2017). The feasibility of automated eye tracking with the Early Childhood Vigilance Test of attention in younger HIV-exposed Ugandan children. *Neuropsychology, 31*(5), 525-534. doi:10.1037/neu0000382

Boo, F. L., Mateus, M. C., & Duryea, S. (2018). Analysis of socioeconomic gradients in the development of children aged 0-3 years in Fortaleza, Northeastern Brazil. *Rev Saude Publica, 52*, 84. doi:10.11606/S1518-8787.2018052000525

Bornstein, M. H., & Putnick, D. L. (2012). Cognitive and socioemotional caregiving in developing countries. *Child Dev, 83*(1), 46-61. doi:10.1111/j.1467-8624.2011.01673.x

Bornstein, M. H., Putnick, D. L., Cote, L. R., Haynes, O. M., & Suwalsky, J. T. (2015). Mother-infant contingent vocalizations in 11 countries. *Psychological Science, 26*(8), 1272-1284.

Bornstein, M. H., Putnick, D. L., Lansford, J. E., Deater-Deckard, K., & Bradley, R. H. (2015). A Developmental Analysis of Caregiving Modalities Across Infancy in 38 Low- and Middle-Income Countries. *Child Dev, 86*(5), 1571-1587. doi:10.1111/cdev.12402

Bougma, K., Aboud, F. E., Lemma, T. M., Frongillo, E. A., & Marquis, G. S. (2018). Introduction of iodised salt benefits infants' mental development in a community-based cluster-randomised effectiveness trial in Ethiopia. *Br J Nutr, 119*(7), 801-809. doi:10.1017/S0007114517003658

Brentani, A., Walker, S., Chang-Lopez, S., Grisi, S., Powell, C., & Fink, G. (2021). A home visit-based early childhood stimulation programme in Brazil-a randomized controlled trial. *Health Policy Plan, 36*(3), 288-297. doi:10.1093/heapol/czaa195

Bryant, J. H., Bryant, N. H., Williams, S., Ndambuki, R. N., & Erwin, P. C. (2012). Addressing social determinants of health by integrating assessment of caregiver-child attachment into community based primary health care in urban Kenya. *Int J Environ Res Public Health, 9*(10), 3588-3598. doi:10.3390/ijerph9103588

Camargo-Figuera, F. A., Barros, A. J., Santos, I. S., Matijasevich, A., & Barros, F. C. (2014). Early life determinants of low IQ at age 6 in children from the 2004 Pelotas Birth Cohort: a predictive approach. *BMC Pediatr, 14*, 308. doi:10.1186/s12887-014-0308-1

Celhay, P., Martinez, S., & Vidal, C. (2020). Measuring socioeconomic gaps in nutrition and early child development in Bolivia. *Int J Equity Health, 19*(1), 122. doi:10.1186/s12939-020-01197-1

Chandna, J., Ntozini, R., Evans, C., Kandawasvika, G., Chasekwa, B., Majo, F., . . . Gladstone, M. (2020). Effects of improved complementary feeding and improved water, sanitation and hygiene on early child development among HIV-exposed children: substudy of a cluster randomised trial in rural Zimbabwe. *BMJ Glob Health, 5*(1), e001718. doi:10.1136/bmjgh-2019-001718

Chang, S. M., Grantham-McGregor, S. M., Powell, C. A., Vera-Hernández, M., Lopez-Boo, F., Baker-Henningham, H., & Walker, S. P. (2015). Integrating a Parenting Intervention With Routine Primary Health Care: A Cluster Randomized Trial. *Pediatrics, 136*(2), 272-280. doi:10.1542/peds.2015-0119

Cheng, N., Lu, S., Archer, M., & Wang, Z. (2018). Quality of Maternal Parenting of 9-Month-Old Infants Predicts Executive Function Performance at 2 and 3 Years of Age. *Front Psychol, 8*, 2293. doi:10.3389/fpsyg.2017.02293

Chingono, R., Mebrahtu, H., Mupambireyi, Z., Simms, V., Weiss, H. A., Ndlovu, P., . . . Sherr, L. (2018). Evaluating the effectiveness of a multi-component intervention on early childhood development in paediatric HIV care and treatment programmes: a randomised controlled trial. *BMC Pediatr, 18*(1), 222. doi:10.1186/s12887-018-1201-0

Chung, E. O., Hagaman, A., LeMasters, K., Andrabi, N., Baranov, V., Bates, L. M., . . . Maselko, J. (2020). The contribution of grandmother involvement to child growth and development: an observational study in rural Pakistan. *BMJ Glob Health, 5*(8). doi:10.1136/bmjgh-2019-002181

Cooper, P. J., Tomlinson, M., Swartz, L., Landman, M., Molteno, C., Stein, A., . . . Murray, L. (2009). Improving quality of mother-infant relationship and infant attachment in socioeconomically deprived community in South Africa: randomised controlled trial. *BMJ, 338*, b974. doi:10.1136/bmj.b974

Cuartas, J., McCoy, D. C., Rey-Guerra, C., Britto, P. R., Beatriz, E., & Salhi, C. (2019). Early childhood exposure to non-violent discipline and physical and psychological aggression in low- and middle-income countries: National, regional, and global prevalence estimates. *Child Abuse Negl, 92*, 93-105. doi:10.1016/j.chiabu.2019.03.021

da Rocha Neves, K., de Souza Morais, R. L., Teixeira, R. A., & Pinto, P. A. (2016). Growth and development and their environmental and biological determinants. *J Pediatr (Rio J), 92*(3), 241-250. doi:10.1016/j.jped.2015.08.007

Dabar, D., Das, R., Nagesh, S., Yadav, V., & Mangal, A. (2016). A Community-based Study on Growth and Development of Under-Five Children in an Urbanized Village of South Delhi. *J Trop Pediatr, 62*(6), 446-456. doi:10.1093/tropej/fmw026

Dagvadorj, A., Ganbaatar, D., O, O. B., Yonemoto, N., Bavuusuren, B., Takehara, K., . . . Akahira-Azuma, M. (2018). Maternal socio-demographic and psychological predictors for risk of developmental delays among young children in Mongolia. *BMC Pediatr, 18*(1), 68. doi:10.1186/s12887-018-1017-y

Davies, L., Dunn, M., Chersich, M., Urban, M., Chetty, C., Olivier, L., & Viljoen, D. (2011). Developmental delay of infants and young children with and without fetal alcohol spectrum disorder in the Northern Cape Province, South Africa. *Afr J Psychiatry (Johannesbg), 14*(4), 298-305. doi:10.4314/ajpsy.v14i4.7

de Castro, F., Place, J. M., Villalobos, A., Rojas, R., Barrientos, T., & Frongillo, E. A. (2017). Poor early childhood outcomes attributable to maternal depression in Mexican women. *Arch Womens Ment Health, 20*(4), 561-568. doi:10.1007/s00737-017-0736-7

de Moura, D. R., Costa, J. C., Santos, I. S., Barros, A. J., Matijasevich, A., Halpern, R., . . . Barros, F. C. (2010). Risk factors for suspected developmental delay at age 2 years in a Brazilian birth cohort. *Paediatr Perinat Epidemiol, 24*(3), 211-221. doi:10.1111/j.1365-3016.2010.01115.x

Dearden, K. A., Hilton, S., Bentley, M. E., Caulfield, L. E., Wilde, C., Ha, P. B., & Marsh, D. (2009). Caregiver verbal encouragement increases food acceptance among Vietnamese toddlers. *J Nutr, 139*(7), 1387-1392. doi:10.3945/jn.108.102780

Diab, S. Y., Isosävi, S., Qouta, S. R., Kuittinen, S., & Punamäki, R. L. (2018). The protective role of maternal posttraumatic growth and cognitive trauma processing among Palestinian mothers and infants. *Infant Behav Dev, 50*, 284-299. doi:10.1016/j.infbeh.2017.05.008

Diniz, E., DeSousa, D., Koller, S. H., & Volling, B. L. (2016). Longitudinal effects of contextual and proximal factors on mother-infant interactions among Brazilian adolescent mothers. *Infant Behav Dev, 43*, 36-43. doi:10.1016/j.infbeh.2016.02.002

Do, C. H. T., Kruse, A. Y., Wills, B., Sabanathan, S., Clapham, H., Pedersen, F. K., . . . Børresen, M. L. (2020). Neurodevelopment at 2 years corrected age among Vietnamese preterm infants. *Arch Dis Child, 105*(2), 134-140. doi:10.1136/archdischild-2019-316967

Donald, K. A., Hoogenhout, M., du Plooy, C. P., Wedderburn, C. J., Nhapi, R. T., Barnett, W., . . . Stein, D. J. (2018). Drakenstein Child Health Study (DCHS): investigating determinants of early child development and cognition. *BMJ Paediatr Open, 2*(1), e000282. doi:10.1136/bmjpo-2018-000282

Donald, K. A., Wedderburn, C. J., Barnett, W., Nhapi, R. T., Rehman, A. M., Stadler, J. A. M., . . . Stein, D. J. (2019). Risk and protective factors for child development: An observational South African birth cohort. *PLoS Med, 16*(9), e1002920. doi:10.1371/journal.pmed.1002920

Dora, B., & Baydar, N. (2020). Transactional associations of maternal depressive symptoms with child externalizing behaviors are small after age 3. *Dev Psychopathol, 32*(1), 293-308. doi:10.1017/S0954579419000075

Drago, F., Scharf, R. J., Maphula, A., Nyathi, E., Mahopo, T. C., Svensen, E., . . . Rogawski McQuade, E. T. (2020). Psychosocial and environmental determinants of child cognitive development in rural south africa and tanzania: findings from the mal-ed cohort. *BMC Public Health, 20*(1), 505. doi:10.1186/s12889-020-08598-5

du Toit, M., van der Linde, J., & Swanepoel, W. (2020). Early Childhood Development Risks and Protective Factors in Vulnerable Preschool Children from Low-Income Communities in South Africa. *J Community Health*. doi:10.1007/s10900-020-00883-z

Duc, N. H. (2016). Developmental risk factors in Vietnamese preschool-age children: Cross-sectional survey. *Pediatr Int, 58*(1), 14-21. doi:10.1111/ped.12748

Eickmann, S. H., de Lira, P. I., Lima Mde, C., Coutinho, S. B., Teixeira Mde, L., & Ashworth, A. (2007). Breast feeding and mental and motor development at 12 months in a low-income population in northeast Brazil. *Paediatr Perinat Epidemiol, 21*(2), 129-137. doi:10.1111/j.1365-3016.2007.00795.x

Ertem, I. O., Atay, G., Bingoler, B. E., Dogan, D. G., Bayhan, A., & Sarica, D. (2006). Promoting child development at sick-child visits: a controlled trial. *Pediatrics, 118*(1), e124-131. doi:10.1542/peds.2005-2704

Familiar, I., Collins, S. M., Sikorskii, A., Ruisenor-Escudero, H., Natamba, B., Bangirana, P., . . . Young, S. L. (2018). Quality of Caregiving is Positively Associated With Neurodevelopment During the First Year of Life Among HIV-Exposed Uninfected Children in Uganda. *J Acquir Immune Defic Syndr, 77*(3), 235-242. doi:10.1097/QAI.0000000000001599

Fatori, D., Argeu, A., Brentani, H., Chiesa, A., Fracolli, L., Matijasevich, A., . . . Polanczyk, G. (2020). Maternal Parenting Electronic Diary in the Context of a Home Visit Intervention for Adolescent Mothers in an Urban Deprived Area of São Paulo, Brazil: Randomized Controlled Trial. *JMIR Mhealth Uhealth, 8*(7), e13686. doi:10.2196/13686

Feng, Y., Zhou, H., Zhang, Y., Perkins, A., Wang, Y., & Sun, J. (2018). Comparison in executive function in Chinese preterm and full-term infants at eight months. *Front Med, 12*(2), 164-173. doi:10.1007/s11684-017-0540-9

Fernald, L. C., Gertler, P. J., & Neufeld, L. M. (2008). Role of cash in conditional cash transfer programmes for child health, growth, and development: an analysis of Mexico's Oportunidades. *Lancet, 371*(9615), 828-837. doi:10.1016/S0140-6736(08)60382-7

Fernald, L. C., & Hidrobo, M. (2011). Effect of Ecuador's cash transfer program (Bono de Desarrollo Humano) on child development in infants and toddlers: a randomized effectiveness trial. *Soc Sci Med, 72*(9), 1437-1446. doi:10.1016/j.socscimed.2011.03.005

Fernald, L. C., Jones-Smith, J. C., Ozer, E. J., Neufeld, L. M., & DiGirolamo, A. M. (2008). Maternal depressive symptoms and physical activity in very low-income children. *J Dev Behav Pediatr, 29*(5), 385-393. doi:10.1097/DBP.0b013e318182a98e

Fernald, L. C., Kariger, P., Hidrobo, M., & Gertler, P. J. (2012). Socioeconomic gradients in child development in very young children: evidence from India, Indonesia, Peru, and Senegal. *Proc Natl Acad Sci U S A, 109 Suppl 2*(Suppl 2), 17273-17280. doi:10.1073/pnas.1121241109

Fink, G., McCoy, D. C., & Yousafzai, A. (2020). Contextual and socioeconomic variation in early motor and language development. *Arch Dis Child, 105*(5), 421-427. doi:10.1136/archdischild-2019-317849

Frith, A. L., Naved, R. T., Persson, L. A., Rasmussen, K. M., & Frongillo, E. A. (2012). Early participation in a prenatal food supplementation program ameliorates the negative association of food insecurity with quality of maternal-infant interaction. *J Nutr, 142*(6), 1095-1101. doi:10.3945/jn.111.155358

Frongillo, E. A., Kulkarni, S., Basnet, S., & de Castro, F. (2017). Family Care Behaviors and Early Childhood Development in Low- and Middle-Income Countries. *Journal of Child and Family Studies, 26*(11), 3036-3044. doi:10.1007/s10826-017-0816-3

Frongillo, E. A., Nguyen, P. H., Saha, K. K., Sanghvi, T., Afsana, K., Haque, R., . . . Menon, P. (2017). Large-Scale Behavior-Change Initiative for Infant and Young Child Feeding Advanced Language and Motor Development in a Cluster-Randomized Program Evaluation in Bangladesh. *J Nutr, 147*(2), 256-263. doi:10.3945/jn.116.240861

Galasso, E., Weber, A. M., Stewart, C. P., Ratsifandrihamanana, L., & Fernald, L. C. H. (2019). Effects of nutritional supplementation and home visiting on growth and development in young children in Madagascar: a cluster-randomised controlled trial. *Lancet Glob Health, 7*(9), e1257-e1268. doi:10.1016/S2214-109X(19)30317-1

Geletu, A., Lelisa, A., & Baye, K. (2019). Provision of low-iron micronutrient powders on alternate days is associated with lower prevalence of anaemia, stunting, and improved motor milestone acquisition in the first year of life: A retrospective cohort study in rural Ethiopia. *Matern Child Nutr, 15*(3), e12785. doi:10.1111/mcn.12785

Gelli, A., Margolies, A., Santacroce, M., Roschnik, N., Twalibu, A., Katundu, M., . . . Ruel, M. (2018). Using a Community-Based Early Childhood Development Center as a Platform to Promote Production and Consumption Diversity Increases Children's Dietary Intake and Reduces Stunting in Malawi: A Cluster-Randomized Trial. *J Nutr, 148*(10), 1587-1597. doi:10.1093/jn/nxy148

Gelli, A., Nguyen, P. H., Santacroce, M., Twalibu, A., Margolies, A., & Katundu, M. (2020). A Community-Based Early Childhood Development Center Platform Promoting Diversified Diets and Food Production Increases the Mean Probability of Adequacy of Intake of Preschoolers in Malawi: A Cluster Randomized Trial. *J Nutr, 150*(2), 350-355. doi:10.1093/jn/nxz245

Gladstone, M. J., Chandna, J., Kandawasvika, G., Ntozini, R., Majo, F. D., Tavengwa, N. V., . . . Prendergast, A. J. (2019). Independent and combined effects of improved water, sanitation, and hygiene (WASH) and improved complementary feeding on early neurodevelopment among children born to HIV-negative mothers in rural Zimbabwe: Substudy of a cluster-randomized trial. *PLoS Med, 16*(3), e1002766. doi:10.1371/journal.pmed.1002766

Grantham-McGregor, S., Adya, A., Attanasio, O., Augsburg, B., Behrman, J., Caeyers, B., . . . Vats, K. (2020). Group Sessions or Home Visits for Early Childhood Development in India: A Cluster RCT. *Pediatrics, 146*(6). doi:10.1542/peds.2020-002725

Hamadani, J. D., Mehrin, S. F., Tofail, F., Hasan, M. I., Huda, S. N., Baker-Henningham, H., . . . Grantham-McGregor, S. (2019). Integrating an early childhood development programme into Bangladeshi primary health-care services: an open-label, cluster-randomised controlled trial. *Lancet Glob Health, 7*(3), e366-e375. doi:10.1016/S2214-109X(18)30535-7

Hamadani, J. D., Tofail, F., Hilaly, A., Huda, S. N., Engle, P., & Grantham-McGregor, S. M. (2010). Use of family care indicators and their relationship with child development in Bangladesh. *J Health Popul Nutr, 28*(1), 23-33. doi:10.3329/jhpn.v28i1.4520

Hamadani, J. D., Tofail, F., Huda, S. N., Alam, D. S., Ridout, D. A., Attanasio, O., & Grantham-McGregor, S. M. (2014). Cognitive deficit and poverty in the first 5 years of childhood in Bangladesh. *Pediatrics, 134*(4), e1001-1008. doi:10.1542/peds.2014-0694

Handal, A. J., Garcia Saavedra, L., Schrader, R., Aragón, C. L., Páez, M., & Lowe, J. R. (2017). Assessment of Maternal-Infant Interaction: Application of the Still Face Paradigm in a Rural Population of Working Women in Ecuador. *Matern Child Health J, 21*(3), 458-466. doi:10.1007/s10995-016-2123-9

Handal, A. J., Lozoff, B., Breilh, J., & Harlow, S. D. (2007a). Effect of community of residence on neurobehavioral development in infants and young children in a flower-growing region of Ecuador. *Environ Health Perspect, 115*(1), 128-133. doi:10.1289/ehp.9261

Handal, A. J., Lozoff, B., Breilh, J., & Harlow, S. D. (2007b). Neurobehavioral development in children with potential exposure to pesticides. *Epidemiology, 18*(3), 312-320. doi:10.1097/01.ede.0000259983.55716.bb

Handal, A. J., Lozoff, B., Breilh, J., & Harlow, S. D. (2007c). Sociodemographic and nutritional correlates of neurobehavioral development: a study of young children in a rural region of Ecuador. *Rev Panam Salud Publica, 21*(5), 292-300. doi:10.1590/s1020-49892007000400004

Hanieh, S., Ha, T. T., Simpson, J. A., Thuy, T. T., Khuong, N. C., Thoang, D. D., . . . Biggs, B. A. (2014). Maternal vitamin D status and infant outcomes in rural Vietnam: a prospective cohort study. *PLoS One, 9*(6), e99005. doi:10.1371/journal.pone.0099005

Hartinger, S. M., Lanata, C. F., Hattendorf, J., Gil, A. I., Verastegui, H., Ochoa, T., & Mausezahl, D. (2011). A community randomised controlled trial evaluating a home-based environmental intervention package of improved stoves, solar water disinfection and kitchen sinks in rural Peru: rationale, trial design and baseline findings. *Contemp Clin Trials, 32*(6), 864-873. doi:10.1016/j.cct.2011.06.006

Hartinger, S. M., Lanata, C. F., Hattendorf, J., Verastegui, H., Gil, A. I., Wolf, J., & Mausezahl, D. (2016). Improving household air, drinking water and hygiene in rural Peru: a community-randomized-controlled trial of an integrated environmental home-based intervention package to improve child health. *Int J Epidemiol, 45*(6), 2089-2099. doi:10.1093/ije/dyw242

Hartinger, S. M., Lanata, C. F., Hattendorf, J., Wolf, J., Gil, A. I., Obando, M. O., . . . Mausezahl, D. (2017). Impact of a child stimulation intervention on early child development in rural Peru: a cluster randomised trial using a reciprocal control design. *J Epidemiol Community Health, 71*(3), 217-224. doi:10.1136/jech-2015-206536

Hollowell, J., Belem, M., Swigart, T., Murray, J., & Hill, Z. (2020). Age-related patterns of early childhood development practices amongst rural families in Burkina Faso: findings from a nationwide survey of mothers of children aged 0-3 years. *Glob Health Action, 13*(1), 1772560. doi:10.1080/16549716.2020.1772560

Hua, J., Duan, T., Gu, G., Wo, D., Zhu, Q., Liu, J. Q., . . . Meng, W. (2016). Effects of home and education environments on children's motor performance in China. *Dev Med Child Neurol, 58*(8), 868-876. doi:10.1111/dmcn.13073

Huiracocha-Tutiven, L., Orellana-Paucar, A., Abril-Ulloa, V., Huiracocha-Tutiven, M., Palacios-Santana, G., & Blume, S. (2019). Child Development and Nutritional Status in Ecuador. *Glob Pediatr Health, 6*, 2333794X18821946. doi:10.1177/2333794X18821946

Huynh, D. T. T., Tran, N. T., Nguyen, L. T., Berde, Y., & Low, Y. L. (2018). Impact of maternal nutritional supplementation in conjunction with a breastfeeding support program on breastfeeding performance, birth, and growth outcomes in a Vietnamese population. *J Matern Fetal Neonatal Med, 31*(12), 1586-1594. doi:10.1080/14767058.2017.1320984

Ip, P., Tso, W., Rao, N., Ho, F. K. W., Chan, K. L., Fu, K. W., . . . Chow, C. B. (2018). Rasch validation of the Chinese parent-child interaction scale (CPCIS). *World J Pediatr, 14*(3), 238-246. doi:10.1007/s12519-018-0132-z

Jensen, S. K. G., Tofail, F., Haque, R., Petri, W. A., Jr., & Nelson, C. A., 3rd. (2019). Child development in the context of biological and psychosocial hazards among poor families in Bangladesh. *PLoS One, 14*(5), e0215304. doi:10.1371/journal.pone.0215304

Jeong, J., Adhia, A., Bhatia, A., McCoy, D. C., & Yousafzai, A. K. (2020). Intimate Partner Violence, Maternal and Paternal Parenting, and Early Child Development. *Pediatrics, 145*(6). doi:10.1542/peds.2019-2955

Jeong, J., Bhatia, A., & Fink, G. (2018). Associations between birth registration and early child growth and development: evidence from 31 low- and middle-income countries. *BMC Public Health, 18*(1), 673. doi:10.1186/s12889-018-5598-z

Jeong, J., McCoy, D. C., Yousafzai, A. K., Salhi, C., & Fink, G. (2016). Paternal Stimulation and Early Child Development in Low- and Middle-Income Countries. *Pediatrics, 138*(4). doi:10.1542/peds.2016-1357

Jeong, J., Siyal, S., & Yousafzai, A. K. (2019). Agreement between Fathers' and Mothers' Reported Stimulation and Associations with Observed Responsive Parenting in Pakistan. *Children (Basel), 6*(10). doi:10.3390/children6100114

Kang, Y., Aguayo, V. M., Campbell, R. K., & West, K. P., Jr. (2018). Association between stunting and early childhood development among children aged 36-59 months in South Asia. *Matern Child Nutr, 14 Suppl 4*(Suppl 4), e12684. doi:10.1111/mcn.12684

Karabekiroglu, K., Uslu, R., Kapci-Seyitoglu, E. G., Özbaran, B., Öztop, D. B., Özel-Özcan, Ö., . . . Cengel-Kültür, E. S. (2013). A nationwide study of social-emotional problems in young children in Turkey. *Infant Behav Dev, 36*(1), 162-170. doi:10.1016/j.infbeh.2012.11.007

Kariger, P., Frongillo, E. A., Engle, P., Britto, P. M., Sywulka, S. M., & Menon, P. (2012). Indicators of family care for development for use in multicountry surveys. *J Health Popul Nutr, 30*(4), 472-486. doi:10.3329/jhpn.v30i4.13417

Kashala-Abotnes, E., Sombo, M. T., Okitundu, D. L., Kunyu, M., Bumoko Makila-Mabe, G., Tylleskär, T., . . . Boivin, M. J. (2018). Dietary cyanogen exposure and early child neurodevelopment: An observational study from the Democratic Republic of Congo. *PLoS One, 13*(4), e0193261. doi:10.1371/journal.pone.0193261

Khandelwal, N., Mandliya, J., Nigam, K., Patil, V., Mathur, A., & Pathak, A. (2020). Determinants of motor, language, cognitive, and global developmental delay in children with complicated severe acute malnutrition at the time of discharge: An observational study from Central India. *PLoS One, 15*(6), e0233949. doi:10.1371/journal.pone.0233949

Khowaja, Y., Karmaliani, R., Hirani, S., Khowaja, A. R., Rafique, G., & McFarlane, J. (2015). A Pilot Study of a 6-Week Parenting Program for Mothers of Pre-school Children Attending Family Health Centers in Karachi, Pakistan. *Int J Health Policy Manag, 5*(2), 91-97. doi:10.15171/ijhpm.2015.181

Knauer, H. A., Jakiela, P., Ozier, O., Aboud, F., & Fernald, L. C. H. (2019). Enhancing young children’s language acquisition through parent–child book-sharing: A randomized trial in rural Kenya. *Early Childhood Research Quarterly, 50*, 179-190. doi:<https://doi.org/10.1016/j.ecresq.2019.01.002>

Knauer, H. A., Ozer, E. J., Dow, W., & Fernald, L. C. H. (2017). Stimulating Parenting Practices in Indigenous and Non-Indigenous Mexican Communities. *Int J Environ Res Public Health, 15*(1). doi:10.3390/ijerph15010029

Koshy, B., Srinivasan, M., Zachariah, S. M., Karthikeyan, A. S., Roshan, R., Bose, A., . . . Kang, G. (2020). Body iron and lead status in early childhood and its effects on development and cognition: a longitudinal study from urban Vellore. *Public Health Nutr, 23*(11), 1896-1906. doi:10.1017/S1368980019004622

Koura, K. G., Boivin, M. J., Davidson, L. L., Ouédraogo, S., Zoumenou, R., Alao, M. J., . . . Bodeau-Livinec, F. (2013). Usefulness of child development assessments for low-resource settings in francophone Africa. *J Dev Behav Pediatr, 34*(7), 486-493. doi:10.1097/DBP.0b013e31829d211c

Kumar, R., Aggarwal, A. K., Kaur, M., & Iyengar, S. D. (1997). Factors influencing psychosocial development of preschool children in a rural area of Haryana, India. *J Trop Pediatr, 43*(6), 324-329. doi:10.1093/tropej/43.6.324

Kumwenda, C., Hemsworth, J., Phuka, J., Ashorn, U., Arimond, M., Maleta, K., . . . Ashorn, P. (2018). Association between breast milk intake at 9-10 months of age and growth and development among Malawian young children. *Matern Child Nutr, 14*(3), e12582. doi:10.1111/mcn.12582

Kvestad, I., Taneja, S., Hysing, M., Kumar, T., Bhandari, N., & Strand, T. A. (2015). Diarrhea, stimulation and growth predict neurodevelopment in young North Indian children. *PLoS One, 10*(3), e0121743. doi:10.1371/journal.pone.0121743

Lahti, K., Vänskä, M., Qouta, S. R., Diab, S. Y., Perko, K., & Punamäki, R. L. (2019). Maternal experience of their infants' crying in the context of war trauma: Determinants and consequences. *Infant Ment Health J, 40*(2), 186-203. doi:10.1002/imhj.21768

Larson, L. M., Martorell, R., & Bauer, P. J. (2018). A Path Analysis of Nutrition, Stimulation, and Child Development Among Young Children in Bihar, India. *Child Dev, 89*(5), 1871-1886. doi:10.1111/cdev.13057

Larson, L. M., Young, M. F., Bauer, P. J., Mehta, R., Girard, A. W., Ramakrishnan, U., . . . Martorell, R. (2018). Effectiveness of a home fortification programme with multiple micronutrients on infant and young child development: a cluster-randomised trial in rural Bihar, India. *Br J Nutr, 120*(2), 176-187. doi:10.1017/S000711451800140X

Larson, L. M., Young, M. F., Ramakrishnan, U., Webb Girard, A., Verma, P., Chaudhuri, I., . . . Martorell, R. (2017). A Cross-Sectional Survey in Rural Bihar, India, Indicates That Nutritional Status, Diet, and Stimulation Are Associated with Motor and Mental Development in Young Children. *J Nutr, 147*(8), 1578-1585. doi:10.3945/jn.117.251231

Laurenzi, C. A., Skeen, S., Sundin, P., Hunt, X., Weiss, R. E., Rotheram-Borus, M. J., & Tomlinson, M. (2020). Associations between young children's exposure to household violence and behavioural problems: Evidence from a rural Kenyan sample. *Glob Public Health, 15*(2), 173-184. doi:10.1080/17441692.2019.1656274

le Roux, S. M., Donald, K. A., Brittain, K., Phillips, T. K., Zerbe, A., Nguyen, K. K., . . . Myer, L. (2018). Neurodevelopment of breastfed HIV-exposed uninfected and HIV-unexposed children in South Africa. *AIDS, 32*(13), 1781-1791. doi:10.1097/QAD.0000000000001872

Li, Z., Jiang, Y., Li, M., & Lu, C. (2018). Inequalities in socio-emotional development and positive parenting during childhood: Evidence from China 2010-2014. *SSM Popul Health, 5*, 8-16. doi:10.1016/j.ssmph.2018.04.007

Lima, M. C., Eickmann, S. H., Lima, A. C., Guerra, M. Q., Lira, P. I., Huttly, S. R., & Ashworth, A. (2004). Determinants of mental and motor development at 12 months in a low income population: a cohort study in northeast Brazil. *Acta Paediatr, 93*(7), 969-975. doi:10.1111/j.1651-2227.2004.tb18257.x

Locks, L. M., Nanama, S., Addo, O. Y., Albert, B., Sandalinas, F., Nanema, A., . . . Tripp, K. (2019). An integrated infant and young child feeding and small-quantity lipid-based nutrient supplementation programme in the Democratic Republic of Congo is associated with improvements in breastfeeding and handwashing behaviours but not dietary diversity. *Matern Child Nutr, 15*(3), e12784. doi:10.1111/mcn.12784

Lozoff, B., Smith, J. B., Clark, K. M., Perales, C. G., Rivera, F., & Castillo, M. (2010). Home intervention improves cognitive and social-emotional scores in iron-deficient anemic infants. *Pediatrics, 126*(4), e884-894. doi:10.1542/peds.2009-3535

Lu, C., Cuartas, J., Fink, G., McCoy, D., Liu, K., Li, Z., . . . Richter, L. (2020). Inequalities in early childhood care and development in low/middle-income countries: 2010-2018. *BMJ Glob Health, 5*(2), e002314. doi:10.1136/bmjgh-2020-002314

Luo, R., Emmers, D., Warrinnier, N., Rozelle, S., & Sylvia, S. (2019). Using community health workers to deliver a scalable integrated parenting program in rural China: A cluster-randomized controlled trial. *Soc Sci Med, 239*, 112545. doi:10.1016/j.socscimed.2019.112545

Luo, R., Shi, Y., Zhou, H., Yue, A., Zhang, L., Sylvia, S., . . . Rozelle, S. (2015). Micronutrient deficiencies and developmental delays among infants: evidence from a cross-sectional survey in rural China. *BMJ Open, 5*(10), e008400. doi:10.1136/bmjopen-2015-008400

Luo, R., Yue, A., Zhou, H., Shi, Y., Zhang, L., Martorell, R., . . . Sylvia, S. (2017). The effect of a micronutrient powder home fortification program on anemia and cognitive outcomes among young children in rural China: a cluster randomized trial. *BMC Public Health, 17*(1), 738. doi:10.1186/s12889-017-4755-0

Luoto, J. E., Lopez Garcia, I., Aboud, F. E., Singla, D. R., Fernald, L. C. H., Pitchik, H. O., . . . Alu, E. (2021). Group-based parenting interventions to promote child development in rural Kenya: a multi-arm, cluster-randomised community effectiveness trial. *Lancet Glob Health, 9*(3), e309-e319. doi:10.1016/S2214-109X(20)30469-1

MacGinty, R. P., Kariuki, S. M., Barnett, W., Wedderburn, C. J., Hardy, A., Hoffman, N., . . . Stein, D. J. (2020). Associations of antenatal maternal psychological distress with infant birth and development outcomes: Results from a South African birth cohort. *Compr Psychiatry, 96*, 152128. doi:10.1016/j.comppsych.2019.152128

Malhi, P., Menon, J., Bharti, B., & Sidhu, M. (2018). Cognitive Development of Toddlers: Does Parental Stimulation Matter? *Indian J Pediatr, 85*(7), 498-503. doi:10.1007/s12098-018-2613-4

Manu, A., Ewerling, F., Barros, A. J., & Victora, C. G. (2019). Association between availability of children's book and the literacy-numeracy skills of children aged 36 to 59 months: secondary analysis of the UNICEF Multiple-Indicator Cluster Surveys covering 35 countries. *J Glob Health, 9*(1), 010403. doi:10.7189/jogh.09.010403

Martinez, B., Cardona, S., Rodas, P., Lubina, M., Gonzalez, A., Farley Webb, M., . . . Rohloff, P. (2018). Developmental outcomes of an individualised complementary feeding intervention for stunted children: a substudy from a larger randomised controlled trial in Guatemala. *BMJ Paediatr Open, 2*(1), e000314. doi:10.1136/bmjpo-2018-000314

Martinez, B., Webb, M. F., Gonzalez, A., Douglas, K., Grazioso, M. D. P., & Rohloff, P. (2018). Complementary feeding intervention on stunted Guatemalan children: a randomised controlled trial. *BMJ Paediatr Open, 2*(1), e000213. doi:10.1136/bmjpo-2017-000213

Maselko, J., Hagaman, A. K., Bates, L. M., Bhalotra, S., Biroli, P., Gallis, J. A., . . . Rahman, A. (2019). Father involvement in the first year of life: Associations with maternal mental health and child development outcomes in rural Pakistan. *Soc Sci Med, 237*, 112421. doi:10.1016/j.socscimed.2019.112421

Mathieu, I., Wallis, K., Japa, I., Cordero, R., Deverlis, A., Steenhoff, A. P., & Lowenthal, E. (2020). Caregiver Strengths, Attitudes, and Concerns About Reading and Child Development in the Dominican Republic. *Glob Pediatr Health, 7*, 2333794X20942661. doi:10.1177/2333794X20942661

Mazzucchelli, T. G., Hodges, J., Kane, R. T., Sofronoff, K., Sanders, M. R., Einfeld, S., . . . Team, M. P. (2018). Parenting and family adjustment scales (PAFAS): validation of a brief parent-report measure for use with families who have a child with a developmental disability. *Res Dev Disabil, 72*, 140-151. doi:10.1016/j.ridd.2017.10.011

McCoy, D. C., Sudfeld, C. R., Bellinger, D. C., Muhihi, A., Ashery, G., Weary, T. E., . . . Fink, G. (2017). Development and validation of an early childhood development scale for use in low-resourced settings. *Popul Health Metr, 15*(1), 3. doi:10.1186/s12963-017-0122-8

McDonald, C. M., Manji, K. P., Kupka, R., Bellinger, D. C., Spiegelman, D., Kisenge, R., . . . Duggan, C. P. (2013). Stunting and wasting are associated with poorer psychomotor and mental development in HIV-exposed Tanzanian infants. *J Nutr, 143*(2), 204-214. doi:10.3945/jn.112.168682

Mebrahtu, H., Simms, V., Mupambireyi, Z., Rehman, A. M., Chingono, R., Matsikire, E., . . . Sherr, L. (2019). Effects of parenting classes and economic strengthening for caregivers on the cognition of HIV-exposed infants: a pragmatic cluster randomised controlled trial in rural Zimbabwe. *BMJ Glob Health, 4*(5), e001651. doi:10.1136/bmjgh-2019-001651

Miller, A. C., Garchitorena, A., Rabemananjara, F., Cordier, L., Randriamanambintsoa, M., Rabeza, V., . . . Ratsifandrihamanana, L. (2020). Factors associated with risk of developmental delay in preschool children in a setting with high rates of malnutrition: a cross-sectional analysis of data from the IHOPE study, Madagascar. *BMC Pediatr, 20*(1), 108. doi:10.1186/s12887-020-1985-6

Miller, A. C., Murray, M. B., Thomson, D. R., & Arbour, M. C. (2016). How consistent are associations between stunting and child development? Evidence from a meta-analysis of associations between stunting and multidimensional child development in fifteen low- and middle-income countries. *Public Health Nutr, 19*(8), 1339-1347. doi:10.1017/S136898001500227X

Mireku, M. O., Cot, M., Massougbodji, A., & Bodeau-Livinec, F. (2020). Relationship between Stunting, Wasting, Underweight and Geophagy and Cognitive Function of Children. *J Trop Pediatr*. doi:10.1093/tropej/fmaa009

Morris, J., Jones, L., Berrino, A., Jordans, M. J., Okema, L., & Crow, C. (2012). Does combining infant stimulation with emergency feeding improve psychosocial outcomes for displaced mothers and babies? A controlled evaluation from northern Uganda. *Am J Orthopsychiatry, 82*(3), 349-357. doi:10.1111/j.1939-0025.2012.01168.x

Muhoozi, G. K., Atukunda, P., Diep, L. M., Mwadime, R., Kaaya, A. N., Skaare, A. B., . . . Iversen, P. O. (2017). Nutrition, hygiene, and stimulation education to improve growth, cognitive, language, and motor development among infants in Uganda: A cluster-randomized trial. *Matern Child Nutr, 14*(2), e12527. doi:10.1111/mcn.12527

Muhoozi, G. K., Atukunda, P., Mwadime, R., Iversen, P. O., & Westerberg, A. C. (2016). Nutritional and developmental status among 6- to 8-month-old children in southwestern Uganda: a cross-sectional study. *Food Nutr Res, 60*, 30270. doi:10.3402/fnr.v60.30270

Murray, L., De Pascalis, L., Tomlinson, M., Vally, Z., Dadomo, H., MacLachlan, B., . . . Cooper, P. J. (2016). Randomized controlled trial of a book-sharing intervention in a deprived South African community: effects on carer-infant interactions, and their relation to infant cognitive and socioemotional outcome. *J Child Psychol Psychiatry, 57*(12), 1370-1379. doi:10.1111/jcpp.12605

Nahar, B., Hamadani, J. D., Ahmed, T., Tofail, F., Rahman, A., Huda, S. N., & Grantham-McGregor, S. M. (2009). Effects of psychosocial stimulation on growth and development of severely malnourished children in a nutrition unit in Bangladesh. *Eur J Clin Nutr, 63*(6), 725-731. doi:10.1038/ejcn.2008.44

Nahar, B., Hossain, I., Hamadani, J. D., Ahmed, T., Grantham-McGregor, S., & Persson, L. A. (2015). Effect of a food supplementation and psychosocial stimulation trial for severely malnourished children on the level of maternal depressive symptoms in Bangladesh. *Child Care Health Dev, 41*(3), 483-493. doi:10.1111/cch.12176

Nahar, B., Hossain, M., Mahfuz, M., Islam, M. M., Hossain, M. I., Murray-Kolb, L. E., . . . Ahmed, T. (2020). Early childhood development and stunting: Findings from the MAL-ED birth cohort study in Bangladesh. *Matern Child Nutr, 16*(1), e12864. doi:10.1111/mcn.12864

Nahar, B., Hossain, M. I., Hamadani, J. D., Ahmed, T., Grantham-McGregor, S., & Persson, L. A. (2012). Effects of psychosocial stimulation on improving home environment and child-rearing practices: results from a community-based trial among severely malnourished children in Bangladesh. *BMC Public Health, 12*, 622. doi:10.1186/1471-2458-12-622

Nahar, B., Hossain, M. I., Hamadani, J. D., Ahmed, T., Huda, S. N., Grantham-McGregor, S. M., & Persson, L. A. (2012). Effects of a community-based approach of food and psychosocial stimulation on growth and development of severely malnourished children in Bangladesh: a randomised trial. *Eur J Clin Nutr, 66*(6), 701-709. doi:10.1038/ejcn.2012.13

Obradović, J., Yousafzai, A. K., Finch, J. E., & Rasheed, M. A. (2016). Maternal scaffolding and home stimulation: Key mediators of early intervention effects on children's cognitive development. *Dev Psychol, 52*(9), 1409-1421. doi:10.1037/dev0000182

Ocansey, M. E., Adu-Afarwuah, S., Kumordzie, S. M., Okronipa, H., Young, R. R., Tamakloe, S. M., . . . Prado, E. L. (2019). Prenatal and postnatal lipid-based nutrient supplementation and cognitive, social-emotional, and motor function in preschool-aged children in Ghana: a follow-up of a randomized controlled trial. *Am J Clin Nutr, 109*(2), 322-334. doi:10.1093/ajcn/nqy303

Olney, D. K., Leroy, J. L., Bliznashka, L., & Ruel, M. T. (2019). A Multisectoral Food-Assisted Maternal and Child Health and Nutrition Program Targeted to Women and Children in the First 1000 Days Increases Attainment of Language and Motor Milestones among Young Burundian Children. *J Nutr, 149*(10), 1833-1842. doi:10.1093/jn/nxz133

Özler, B., Fernald, L. C. H., Kariger, P., McConnell, C., Neuman, M., & Fraga, E. (2018). Combining pre-school teacher training with parenting education: A cluster-randomized controlled trial. *Journal of Development Economics, 133*, 448-467. doi:<https://doi.org/10.1016/j.jdeveco.2018.04.004>

Pace, G. T., Lee, S. J., & Grogan-Kaylor, A. (2019). Spanking and young children's socioemotional development in low- and middle-income countries. *Child Abuse Negl, 88*, 84-95. doi:10.1016/j.chiabu.2018.11.003

Park, S., Bellinger, D. C., Adamo, M., Bennett, B., Choi, N. K., Baltazar, P. I., . . . Friedman, J. F. (2016). Mechanistic Pathways From Early Gestation Through Infancy and Neurodevelopment. *Pediatrics, 138*(6). doi:10.1542/peds.2016-1843

Pearson, R. M., Culpin, I., Loret de Mola, C., Matijasevich, A., Santos, I. S., Horta, B. L., . . . Stein, A. (2019). Grandmothers' mental health is associated with grandchildren's emotional and behavioral development: a three-generation prospective study in Brazil. *BMC Psychiatry, 19*(1), 184. doi:10.1186/s12888-019-2166-8

Perez, E. M., Hendricks, M. K., Beard, J. L., Murray-Kolb, L. E., Berg, A., Tomlinson, M., . . . Vernon-Feagans, L. (2005). Mother-infant interactions and infant development are altered by maternal iron deficiency anemia. *J Nutr, 135*(4), 850-855. doi:10.1093/jn/135.4.850

Pitchik, H. O., Fawzi, W. W., McCoy, D. C., Darling, A. M., Abioye, A. I., Tesha, F., . . . Sudfeld, C. R. (2018). Prenatal nutrition, stimulation, and exposure to punishment are associated with early child motor, cognitive, language, and socioemotional development in Dar es Salaam, Tanzania. *Child Care Health Dev, 44*(6), 841-849. doi:10.1111/cch.12605

Ponguta, L. A., Issa, G., Aoudeh, L., Maalouf, C., Hein, S. D., Zonderman, A. L., . . . Leckman, J. F. (2020). Effects of the Mother-Child Education Program on Parenting Stress and Disciplinary Practices Among Refugee and Other Marginalized Communities in Lebanon: A Pilot Randomized Controlled Trial. *J Am Acad Child Adolesc Psychiatry, 59*(6), 727-738. doi:10.1016/j.jaac.2019.12.010

Powell, C., Baker-Henningham, H., Walker, S., Gernay, J., & Grantham-McGregor, S. (2004). Feasibility of integrating early stimulation into primary care for undernourished Jamaican children: cluster randomised controlled trial. *BMJ, 329*(7457), 89. doi:10.1136/bmj.38132.503472.7C

Prado, E. L., Maleta, K., Caswell, B. L., George, M., Oakes, L. M., DeBolt, M. C., . . . Stewart, C. P. (2020). Early Child Development Outcomes of a Randomized Trial Providing 1 Egg Per Day to Children Age 6 to 15 Months in Malawi. *J Nutr, 150*(7), 1933-1942. doi:10.1093/jn/nxaa088

Quevedo, L. A., Silva, R. A., Godoy, R., Jansen, K., Matos, M. B., Tavares Pinheiro, K. A., & Pinheiro, R. T. (2012). The impact of maternal post-partum depression on the language development of children at 12 months. *Child Care Health Dev, 38*(3), 420-424. doi:10.1111/j.1365-2214.2011.01251.x

Rao, N., Richards, B., Lau, C., Weber, A. M., Sun, J., Darmstadt, G. L., . . . Ip, P. (2020). Associations Among Early Stimulation, Stunting, and Child Development in Four Countries in the East Asia–Pacific. *International Journal of Early Childhood, 52*(2), 175-193. doi:10.1007/s13158-020-00270-8

Rasheed, M. A., & Yousafzai, A. K. (2015). The development and reliability of an observational tool for assessing mother-child interactions in field studies- experience from Pakistan. *Child Care Health Dev, 41*(6), 1161-1171. doi:10.1111/cch.12287

Rempel, J. K., Rempel, L. A., Hoa, D. T. P., Vui, L. T., & Long, T. K. (2020). Parenting Teamwork: The Impact of a Fathering Intervention on Mothers and Infants in Vietnam. *Child Dev, 91*(2), e345-e364. doi:10.1111/cdev.13244

Rempel, L. A., Rempel, J. K., Khuc, T. N., & Vui, L. T. (2017). Influence of father-infant relationship on infant development: A father-involvement intervention in Vietnam. *Dev Psychol, 53*(10), 1844-1858. doi:10.1037/dev0000390

Ribe, I. G., Svensen, E., Lyngmo, B. A., Mduma, E., & Hinderaker, S. G. (2018). Determinants of early child development in rural Tanzania. *Child Adolesc Psychiatry Ment Health, 12*, 18. doi:10.1186/s13034-018-0224-5

Rockers, P. C., Fink, G., Zanolini, A., Banda, B., Biemba, G., Sullivan, C., . . . Hamer, D. H. (2016). Impact of a community-based package of interventions on child development in Zambia: a cluster-randomised controlled trial. *BMJ Glob Health, 1*(3), e000104. doi:10.1136/bmjgh-2016-000104

Rockers, P. C., Zanolini, A., Banda, B., Chipili, M. M., Hughes, R. C., Hamer, D. H., & Fink, G. (2018). Two-year impact of community-based health screening and parenting groups on child development in Zambia: Follow-up to a cluster-randomized controlled trial. *PLoS Med, 15*(4), e1002555. doi:10.1371/journal.pmed.1002555

Rodriguez, V. J., Peltzer, K., Matseke, G., Weiss, S. M., Shine, A., & Jones, D. L. (2018). Pre- and postnatal exposure to intimate partner violence among South African HIV-infected mothers and infant developmental functioning at 12 months of age. *Arch Womens Ment Health, 21*(6), 707-713. doi:10.1007/s00737-018-0857-7

Roopnarine, J. L., Jin, B., & Krishnakumar, A. (2014). Do Guyanese mothers' levels of warmth moderate the association between harshness and justness of physical punishment and preschoolers' prosocial behaviours and anger? *Int J Psychol, 49*(4), 271-279. doi:10.1002/ijop.12029

Rubio-Codina, M., Attanasio, O., Meghir, C., Varela, N., & Grantham-McGregor, S. (2015). The socioeconomic gradient of child development: Cross-sectional evidence from children 6–42 months in Bogota. *Journal of Human Resources, 50*(2), 464-483.

Rubio-Codina, M., & Grantham-McGregor, S. (2019). Evolution of the wealth gap in child development and mediating pathways: Evidence from a longitudinal study in Bogota, Colombia. *Dev Sci, 22*(5), e12810. doi:10.1111/desc.12810

Sajedi, F., Habibi, E., Hatamizadeh, N., Shahshahanipour, S., & Malek Afzali, H. (2018). Early storybook reading and childhood development: A cross-sectional study in Iran. *F1000Res, 7*, 411. doi:10.12688/f1000research.14078.1

Salhi, C., Beatriz, E., McBain, R., McCoy, D., Sheridan, M., & Fink, G. (2021). Physical Discipline, Deprivation, and Differential Risk of Developmental Delay Across 17 Countries. *J Am Acad Child Adolesc Psychiatry, 60*(2), 296-306. doi:10.1016/j.jaac.2020.02.016

Samanta, S., Basu, S. S., Haldar, D., Sarkar, A. P., Saren, A. B., & Sarkar, G. N. (2017). Status of early childhood education under integrated child development services scheme in Bankura Municipality, West Bengal. *Indian J Public Health, 61*(4), 261-266. doi:10.4103/ijph.IJPH_369_16

Sangtawesin, V., Singarj, Y., & Kanjanapattanakul, W. (2011). Growth and development of very low birth weight infants aged 18-24 months at Queen Sirikit National Institute of Child Health. *J Med Assoc Thai, 94 Suppl 3*, S101-106.

Sania, A., Sudfeld, C. R., Danaei, G., Fink, G., McCoy, D. C., Zhu, Z., . . . Fawzi, W. (2019). Early life risk factors of motor, cognitive and language development: a pooled analysis of studies from low/middle-income countries. *BMJ Open, 9*(10), e026449. doi:10.1136/bmjopen-2018-026449

Sarkar, D., & Sarkar, J. (2017). What Does Attending Early Childhood Program Mean for Child Health in India? *Health Econ, 26*(11), 1366-1379. doi:10.1002/hec.3423

Scharf, R. J., Rogawski, E. T., Murray-Kolb, L. E., Maphula, A., Svensen, E., Tofail, F., . . . DeBoer, M. D. (2018). Early childhood growth and cognitive outcomes: Findings from the MAL-ED study. *Matern Child Nutr, 14*(3), e12584. doi:10.1111/mcn.12584

Scherer, E., Hagaman, A., Chung, E., Rahman, A., O'Donnell, K., & Maselko, J. (2019). The relationship between responsive caregiving and child outcomes: evidence from direct observations of mother-child dyads in Pakistan. *BMC Public Health, 19*(1), 252. doi:10.1186/s12889-019-6571-1

Schneider, A., Rodrigues, M., Falenchuk, O., Munhoz, T. N., Barros, A. J. D., Murray, J., . . . Jenkins, J. M. (2021). Cross-Cultural Adaptation and Validation of the Brazilian Portuguese Version of an Observational Measure for Parent-Child Responsive Caregiving. *Int J Environ Res Public Health, 18*(3). doi:10.3390/ijerph18031246

Servili, C., Medhin, G., Hanlon, C., Tomlinson, M., Worku, B., Baheretibeb, Y., . . . Prince, M. (2010). Maternal common mental disorders and infant development in Ethiopia: the P-MaMiE Birth Cohort. *BMC Public Health, 10*, 693. doi:10.1186/1471-2458-10-693

Shattnawi, K. K., & Al-Ali, N. (2019). The Effect of Short Duration Skin to Skin Contact on Premature Infants' Physiological and Behavioral Outcomes: A Quasi-Experimental Study. *J Pediatr Nurs, 46*, e24-e28. doi:10.1016/j.pedn.2019.02.005

Shi, H., Li, X., Fang, H., Zhang, J., & Wang, X. (2020). The Effectiveness and Cost-effectiveness of a Parenting Intervention Integrated with Primary Health Care on Early Childhood Development: a Cluster-Randomized Controlled Trial. *Prev Sci, 21*(5), 661-671. doi:10.1007/s11121-020-01126-2

Sikander, S., Ahmad, I., Bates, L. M., Gallis, J., Hagaman, A., O'Donnell, K., . . . Maselko, J. (2019). Cohort Profile: Perinatal depression and child socioemotional development ; the Bachpan cohort study from rural Pakistan. *BMJ Open, 9*(5), e025644. doi:10.1136/bmjopen-2018-025644

Sincovich, A., Gregory, T., Zanon, C., Santos, D. D., Lynch, J., & Brinkman, S. A. (2020). Measuring early child development in low and middle income countries: Investigating the validity of the early Human Capability Index. *SSM Popul Health, 11*, 100613. doi:10.1016/j.ssmph.2020.100613

Singla, D. R., Kumbakumba, E., & Aboud, F. E. (2015). Effects of a parenting intervention to address maternal psychological wellbeing and child development and growth in rural Uganda: a community-based, cluster randomised trial. *Lancet Glob Health, 3*(8), e458-e469. doi:10.1016/S2214-109X(15)00099-6

Smuts, C. M., Matsungo, T. M., Malan, L., Kruger, H. S., Rothman, M., Kvalsvig, J. D., . . . Faber, M. (2019). Effect of small-quantity lipid-based nutrient supplements on growth, psychomotor development, iron status, and morbidity among 6- to 12-mo-old infants in South Africa: a randomized controlled trial. *Am J Clin Nutr, 109*(1), 55-68. doi:10.1093/ajcn/nqy282

Struyf, T., Dube, Q., Cromwell, E. A., Sheahan, A. D., Heyderman, R. S., & Van Rie, A. (2020). The effect of HIV infection and exposure on cognitive development in the first two years of life in Malawi. *Eur J Paediatr Neurol, 25*, 157-164. doi:10.1016/j.ejpn.2019.11.004

Surkan, P. J., Charles, M. K., Katz, J., Siegel, E. H., Khatry, S. K., LeClerq, S. C., . . . Tielsch, J. M. (2015). The role of zinc and iron-folic acid supplementation on early child temperament and eating behaviors in rural Nepal: a randomized controlled trial. *PLoS One, 10*(3), e0114266. doi:10.1371/journal.pone.0114266

Tessier, R., Charpak, N., Giron, M., Cristo, M., de Calume, Z. F., & Ruiz-Peláez, J. G. (2009). Kangaroo Mother Care, home environment and father involvement in the first year of life: a randomized controlled study. *Acta Paediatr, 98*(9), 1444-1450. doi:10.1111/j.1651-2227.2009.01370.x

Till, C., Dudani, A., Córdoba, L., Cano, J. C., Green, R., Menezes-Filho, J. A., . . . van Wendel de Joode, B. (2019). Caregiving and infants' neurodevelopment in rural Costa Rica: Results from the Infants' Environmental Health Study (ISA). *Neurotoxicology, 74*, 100-107. doi:10.1016/j.neuro.2019.06.002

Tofail, F., Hamadani, J. D., Mehrin, F., Ridout, D. A., Huda, S. N., & Grantham-McGregor, S. M. (2013). Psychosocial stimulation benefits development in nonanemic children but not in anemic, iron-deficient children. *J Nutr, 143*(6), 885-893. doi:10.3945/jn.112.160473

Tomlinson, M., Rabie, S., Skeen, S., Hunt, X., Murray, L., & Cooper, P. J. (2020). Improving mother-infant interaction during infant feeding: A randomised controlled trial in a low-income community in South Africa. *Infant Ment Health J, 41*(6), 850-858. doi:10.1002/imhj.21881

Tran, T. D., Luchters, S., & Fisher, J. (2017). Early childhood development: impact of national human development, family poverty, parenting practices and access to early childhood education. *Child Care Health Dev, 43*(3), 415-426. doi:10.1111/cch.12395

Tumwine, J. K., Nankabirwa, V., Diallo, H. A., Engebretsen, I. M. S., Ndeezi, G., Bangirana, P., . . . Meda, N. (2018). Exclusive breastfeeding promotion and neuropsychological outcomes in 5-8 year old children from Uganda and Burkina Faso: Results from the PROMISE EBF cluster randomized trial. *PLoS One, 13*(2), e0191001. doi:10.1371/journal.pone.0191001

Uwemedimo, O. T., Howlader, A., & Pierret, G. (2017). Parenting Practices and Associations with Development Delays among Young Children in Dominican Republic. *Ann Glob Health, 83*(3-4), 568-576. doi:10.1016/j.aogh.2017.10.007

Vazir, S., Engle, P., Balakrishna, N., Griffiths, P. L., Johnson, S. L., Creed-Kanashiro, H., . . . Bentley, M. E. (2013). Cluster-randomized trial on complementary and responsive feeding education to caregivers found improved dietary intake, growth and development among rural Indian toddlers. *Matern Child Nutr, 9*(1), 99-117. doi:10.1111/j.1740-8709.2012.00413.x

Wang, Y., Chen, X., Yu, Y., Liu, Y., Zhang, Q., & Bai, J. (2020). Association between Gut Microbiota and Infant's Temperament in the First Year of Life in a Chinese Birth Cohort. *Microorganisms, 8*(5). doi:10.3390/microorganisms8050753

Weber, A. M., Rubio-Codina, M., Walker, S. P., van Buuren, S., Eekhout, I., Grantham-McGregor, S. M., . . . Black, M. M. (2019). The D-score: a metric for interpreting the early development of infants and toddlers across global settings. *BMJ Glob Health, 4*(6), e001724. doi:10.1136/bmjgh-2019-001724

Wedderburn, C. J., Yeung, S., Rehman, A. M., Stadler, J. A. M., Nhapi, R. T., Barnett, W., . . . Donald, K. A. (2019). Neurodevelopment of HIV-exposed uninfected children in South Africa: outcomes from an observational birth cohort study. *Lancet Child Adolesc Health, 3*(11), 803-813. doi:10.1016/S2352-4642(19)30250-0

Wei, Q., Zhang, C., Zhang, J., Luo, S., & Wang, X. (2018). CAREGIVER'S DEPRESSIVE SYMPTOMS AND YOUNG CHILDREN'S SOCIOEMOTIONAL DEVELOPMENT DELAYS: A CROSS-SECTIONAL STUDY IN POOR RURAL AREAS OF CHINA. *Infant Ment Health J, 39*(2), 209-219. doi:10.1002/imhj.21699

Wei, Q., Zhang, J. X., Scherpbier, R. W., Zhao, C. X., Luo, S. S., Wang, X. L., & Guo, S. F. (2015). High prevalence of developmental delay among children under three years of age in poverty-stricken areas of China. *Public Health, 129*(12), 1610-1617. doi:10.1016/j.puhe.2015.07.036

Weisleder, A., Mazzuchelli, D. S. R., Lopez, A. S., Neto, W. D., Cates, C. B., Gonçalves, H. A., . . . Mendelsohn, A. L. (2018). Reading Aloud and Child Development: A Cluster-Randomized Trial in Brazil. *Pediatrics, 141*(1). doi:10.1542/peds.2017-0723

Wolf, S., & McCoy, D. C. (2019). Household Socioeconomic Status and Parental Investments: Direct and Indirect Relations With School Readiness in Ghana. *Child Dev, 90*(1), 260-278. doi:10.1111/cdev.12899

Worku, B. N., Abessa, T. G., Wondafrash, M., Lemmens, J., Valy, J., Bruckers, L., . . . Granitzer, M. (2018). Effects of home-based play-assisted stimulation on developmental performances of children living in extreme poverty: a randomized single-blind controlled trial. *BMC Pediatr, 18*(1), 29. doi:10.1186/s12887-018-1023-0

Worku, B. N., Abessa, T. G., Wondafrash, M., Vanvuchelen, M., Bruckers, L., Kolsteren, P., & Granitzer, M. (2018). The relationship of undernutrition/psychosocial factors and developmental outcomes of children in extreme poverty in Ethiopia. *BMC Pediatr, 18*(1), 45. doi:10.1186/s12887-018-1009-y

Xing, X., Wang, M., & Wang, Z. (2018). Parental corporal punishment in relation to children's executive function and externalizing behavior problems in China. *Soc Neurosci, 13*(2), 184-189. doi:10.1080/17470919.2016.1258009

Yang, J., Zhang, Y., Li, H., Wang, N., Yan, S., Zhang, F., . . . Zhao, C. (2020). The Possible Effects of Breastfeeding on Infant Development at 3 Months: A Case-Control Study. *Breastfeed Med*. doi:10.1089/bfm.2019.0283

Ye, A., Yan, S., Huang, K., Mao, L., Ge, X., Weng, T., . . . Tao, F. (2019). Maternal intelligence quotient and motor development in early childhood: The mediating role of mother's education. *J Paediatr Child Health, 55*(1), 87-94. doi:10.1111/jpc.14123

Yousafzai, A. K., Obradović, J., Rasheed, M. A., Rizvi, A., Portilla, X. A., Tirado-Strayer, N., . . . Memon, U. (2016). Effects of responsive stimulation and nutrition interventions on children's development and growth at age 4 years in a disadvantaged population in Pakistan: a longitudinal follow-up of a cluster-randomised factorial effectiveness trial. *Lancet Glob Health, 4*(8), e548-558. doi:10.1016/S2214-109X(16)30100-0

Yousafzai, A. K., Rasheed, M. A., Rizvi, A., Armstrong, R., & Bhutta, Z. A. (2014). Effect of integrated responsive stimulation and nutrition interventions in the Lady Health Worker programme in Pakistan on child development, growth, and health outcomes: a cluster-randomised factorial effectiveness trial. *Lancet, 384*(9950), 1282-1293. doi:10.1016/S0140-6736(14)60455-4

Yousafzai, A. K., Rasheed, M. A., Rizvi, A., Armstrong, R., & Bhutta, Z. A. (2015). Parenting Skills and Emotional Availability: An RCT. *Pediatrics, 135*(5), e1247-1257. doi:10.1542/peds.2014-2335

Yue, A., Shi, Y., Luo, R., Wang, B., Weber, A., Medina, A., . . . Rozelle, S. (2019). Stimulation and Early Child Development in China: Caregiving at Arm's Length. *J Dev Behav Pediatr, 40*(6), 458-467. doi:10.1097/DBP.0000000000000678

Zhang, J., Guo, S., Li, Y., Wei, Q., Zhang, C., Wang, X., . . . Scherpbier, R. W. (2018). Factors influencing developmental delay among young children in poor rural China: a latent variable approach. *BMJ Open, 8*(8), e021628. doi:10.1136/bmjopen-2018-021628

Zhang, S., Dang, R., Yang, N., Bai, Y., Wang, L., Abbey, C., & Rozelle, S. (2018). Effect of Caregiver's Mental Health on Early Childhood Development across Different Rural Communities in China. *Int J Environ Res Public Health, 15*(11). doi:10.3390/ijerph15112341

Zhang, Z., Tran, N. T., Nguyen, T. S., Nguyen, L. T., Berde, Y., Tey, S. L., . . . Huynh, D. T. T. (2018). Impact of maternal nutritional supplementation in conjunction with a breastfeeding support program during the last trimester to 12 weeks postpartum on breastfeeding practices and child development at 30 months old. *PLoS One, 13*(7), e0200519. doi:10.1371/journal.pone.0200519

Zhong, J., He, Y., Chen, Y., & Luo, R. (2020). Relationships between Parenting Skills and Early Childhood Development in Rural Households in Western China. *Int J Environ Res Public Health, 17*(5). doi:10.3390/ijerph17051506

Zhong, J., He, Y., Gao, J., Wang, T., & Luo, R. (2020). Parenting Knowledge, Parental Investments, and Early Childhood Development in Rural Households in Western China. *Int J Environ Res Public Health, 17*(8). doi:10.3390/ijerph17082792

Zhong, J., & Luo, R. (2020). Interrelationships of Parenting Information, Family Care, and Child Development: A Cross-sectional Study in Rural China. *Int J Environ Res Public Health, 17*(16). doi:10.3390/ijerph17165737

Zhou, S., Zhao, C., Huang, X., Li, Z., Ye, R., Shi, H., . . . Scherpbier, R. W. (2019). The effect of a community-based, integrated and nurturing care intervention on early childhood development in rural China. *Public Health, 167*, 125-135. doi:10.1016/j.puhe.2018.11.010
