# Supplementary material for: Measurement tools and indicators for assessing nurturing care for early childhood development: A scoping review
Source: PLOS Glob Public Health. 2022 Apr 25;2(4):e0000373. doi: 10.1371/journal.pgph.0000373 (PMC10021181; doi:10.1371/journal.pgph.0000373)
Supplement: Attachment — Submitted filename: Plos Global Public Health reviewer comments.docx [file pgph.0000373.s004.docx]

**Reviewer #1:**

**The authors have done very well in reviewing and delineating the pertinent literature in this scoping review of what is not just a very vast field but also a very important one. The manuscript itself is well written and organized and easy to understand and comprehend.**

- Thank you for your helpful comments and support of our study.
- **Manuscript pg 6; Methods section and search strategy: Please mention the time period during which the review was done**
  - We have added a sentence in the search strategy section that states we reviewed articles published between database inception until November 30, 2020. In the data extraction section, we have added the time period when our team conducted the extraction (October 2020 to January 2021).
- **Table 4: Time or duration of assessment are given for some observational tools but not for all of them. It would be advised that assessment time duration be included for all tools to ensure consistency.**
  - In Table 4, we have added a new column to that indicates the assessment time for observational tools.
- **Page 37: Please rephrase scoring description in Parent-child relationship (OMCI)**
  - We have revised this description.
- **Page 44: Please rephrase scoring description for the entire item for reference number 53.**
  - We have revised this description.
- **Please note that the above mentioned items on pages 37 and 44 have used the same wording as the source text. The authors are advised to revisit the statements and rephrase the description.**
  - We have revised these descriptions.

**Reviewer #2:**

- **[Abstract] The authors state that there has been increasing political interest and evidence on effective programs …… It would be appropriate for the authors to contextualize their study to public health, not otherwise.**
  - We have revised the introduction of the Abstract.
- **In the introduction, the authors mention the five distinct components of nurturing care. However, only four are mentioned. Please mention the fifth one too.**
  - This has been done.
- **The first sentence of the results section fits to be in the methodology section of the abstract.**
  - In the Results section of the Abstract, we have revised the first sentence to present it more clearly as a result (not a sentence about methodology) indicating the number of studies that ultimately met inclusion criteria.
- **In the first line of paragraph 2 of the introduction, the authors specify the SDG(s) and corresponding targets(s) that is/are in line with the argument being put across.**
  - We have specified that the most relevant SDG target is 4.2 which calls for universal access to quality early childhood development, care, and pre-primary education.
- **In the 3rd paragraph, the authors mention about the five strategic action areas that are crucial for creating an enabling environment for families and caregivers to support young children’s development. Please state those strategic actions you are referring to.**
  - We have revised this sentence to draw less attention to the fact that there are five strategic actions, but instead be more specific about the one strategic action most relevant to the present study – that is the strategic action pertaining to “monitoring progress”.
- **It would be of help if you strengthen your introduction with some statistics around the magnitude of the problem.**
  - This has been done in the first paragraph of the introduction.
- **In paragraph 4, you have not been specific enough about the whether the previous (scoping) reviews have only focused on health and nutrition, hence justifying yours which is rather broader.**
  - We have specified that prior evidence reviews have focused mostly on health and nutrition.
- **[Discussion] Line 352: the authors state that most measures assessed the general qualities of parenting rather than specifically responsive caregiving. It could be helpful if some sources justifying this statement are included her.**
  - We have added references to support this statement.
- **Line 376: Authors state that the outcome measures for early learning and responsive caregiving were largely analysed as continuous or index scores. Please cite the relevant sources supporting this statement.**
  - We have added references to support this statement.
- **Generally, in the discussion, only seven (7) sources were cited to back up the arguments. This is inadequate. There is need for authors to search for more relevant sources to back up their arguments.**
  - We have included additional references throughout the discussion to support our arguments.
- **Generally, the authors have done a fair job.**
  - Thank you for your helpful comments and support of our study.

**Reviewer #3:**

**The diversity of methods and indicators for measuring nurturing care has been well described and demonstrated in this manuscript. The authors made an inventory of the tools and indicators used and showed the interest in standardisation. The topic seems relevant to me. The manuscript can be accepted for publication in its current form.**

- Thank you for your helpful comments and support of our study.
- **However, the authors can clarify the criteria used to select 239 of the 3091 studies identified. Perhaps a diagram could better demonstrate this.**
  - Previously we had a PRISMA flow diagram as a supplementary figure (S1 Fig). We have now moved this into the manuscript as a main figure (Figure 1).
- **In lines 349-350, the authors mention greater variation in definition, measures, and indicators used for outcomes of early learning, responsive care giving, and safety and security. How has this been taken into account in this study, particularly in the discussion and limitations of the study?**
  - We have clarified this point in the first paragraph of the Discussion and again in subsequent paragraphs of the Discussion.
